# 5-HT-dependent synaptic plasticity of the prefrontal cortex in postnatal development

**DOI:** 10.1038/s41598-022-23767-9

**Published:** 2022-12-05

**Authors:** Guilherme Shigueto Vilar Higa, José Francis-Oliveira, Estevão Carlos-Lima, Alicia Moraes Tamais, Fernando da Silva Borges, Alexandre Hiroaki Kihara, Ianê Carvalho Shieh, Henning Ulrich, Silvana Chiavegatto, Roberto De Pasquale

**Affiliations:** 1 Departamento de Fisiologia e Biofísica, Laboratório de Neurofisiologia, Universidade de São Paulo (USP), Butantã, São Paulo, SP 05508-000 Brazil; 2 Laboratório de Neurogenética, Universidade Federal do ABC, São Bernardo do Campo, SP 09606-045 Brazil; 3 Postdoctoral research, Department of Physiology & Pharmacology, SUNY Downstate Health Sciences Brooklyn, Brooklyn, NY 11203 USA; 4 Departamento de Bioquímica, Instituto de Química, Universidade de São Paulo, (USP), Butantã, São Paulo, SP 05508-900 Brazil; 5 Laboratório de Neurociência Comportamental e Molecular, Departamento de Farmacologia, Instituto de Ciências Biomédicas (ICB), Universidade de São Paulo (USP), Butantã, São Paulo, SP 05508-000 Brazil; 6 Departamento de Psiquiatria, Instituto de Psiquiatria do Hospital das Clínicas da Faculdade de Medicina da Universidade de São Paulo (HCFMUSP), São Paulo, SP 05508-903 Brazil; 7 PDC, Center for Mathematics Computation and Cognition, Universidade Federal do ABC, São Bernardo do Campo, Brazil

**Keywords:** Neuroscience, Synaptic plasticity, Long-term depression, Long-term potentiation

## Abstract

Important functions of the prefrontal cortex (PFC) are established during early life, when neurons exhibit enhanced synaptic plasticity and synaptogenesis. This developmental stage drives the organization of cortical connectivity, responsible for establishing behavioral patterns. Serotonin (5-HT) emerges among the most significant factors that modulate brain activity during postnatal development. In the PFC, activated 5-HT receptors modify neuronal excitability and interact with intracellular signaling involved in synaptic modifications, thus suggesting that 5-HT might participate in early postnatal plasticity. To test this hypothesis, we employed intracellular electrophysiological recordings of PFC layer 5 neurons to study the modulatory effects of 5-HT on plasticity induced by theta-burst stimulation (TBS) in two postnatal periods of rats. Our results indicate that 5-HT is essential for TBS to result in synaptic changes during the third postnatal week, but not later. TBS coupled with 5-HT_2A_ or 5-HT_1A_ and 5-HT_7_ receptors stimulation leads to long-term depression (LTD). On the other hand, TBS and synergic activation of 5-HT_1A_, 5-HT_2A_, and 5-HT_7_ receptors lead to long-term potentiation (LTP). Finally, we also show that 5-HT dependent synaptic plasticity of the PFC is impaired in animals that are exposed to early-life chronic stress.

## Introduction

The prefrontal cortex (PFC) plays a central role in regulating complex behaviors by acting as a mediator between sensory, emotional, and associative information^1,2^. In rodents, part of PFC development occurs during the first three postnatal weeks, when layer 5 neurons exhibit high intrinsic excitability and enhanced rates of synaptogenesis^3–6^. Studies carried out in this phase identified that excitatory activity leads to the building of new synaptic connections and reorganization of cortical circuits, with critical consequences in adult behavioral phenotype^7–12^. According to these findings, prefrontal neurons undergo a temporal window of higher synaptic modifications in early life. This property could play a key role in shaping primordial circuits that define cortical functions^6,13–15^. In this respect, it has recently been reported that impairment of excitatory activity during early life leads to reduced dendritic arborization, decreased dendritic spine density, and alterations in circuit formation, which are accompanied by cognitive deficits and maladaptive behaviors^11,16^.

Serotonin (5-HT) is one of the most influential neuromodulators regulating PFC activity state and synaptic plasticity throughout the entire lifespan, including in the early life ages^17,18^. In the mammalian brain, serotonergic neurons are among the first cells to differentiate during postnatal development^19^. In the rodent cortex, 5-HT availability by the raphe nuclei critically increases during the first three weeks of postnatal life^20–22^. Such period corresponds to the temporal window of heightened synaptic reorganization in developing cortical neurons^23–28^. These findings suggest the intriguing possibility that high levels of synaptic change during postnatal development can be maintained by 5-HT modulation of synaptic plasticity driven by neural activity in PFC neurons^29^. Nevertheless, related investigations restricted to juvenile animals have indicated different results. Some studies suggest that 5-HT activates 5-HT_1A_ receptors to modulate synaptic plasticity, whose outcome (long-term potentiation, LTP, or long-term depression, LTD) depends on 5-HT_1A_ receptors interaction with different intracellular cascades^30,31^. Other studies instead suggest that 5-HT acts through 5-HT_2A_ receptors, polarizing synaptic modification towards LTD^32,33^. Indirect evidence indicates that 5-HT might facilitate plasticity induced by bioelectrical signals also at earlier stages of postnatal development^29,34^. For instance, 5-HT depolarizes immature PFC pyramidal neurons through the stimulation of 5-HT_2A_ and 5-HT_7_ receptors, which could support the induction of synaptic modifications by promoting the activation of NMDA receptors^29,34^.

Based on these insights, we adopted intracellular electrophysiological recordings of PFC layer 5 neurons to study the effects of serotonergic modulation on synaptic plasticity at two different postnatal periods. Our results show that 5-HT allows the occurrence of synaptic changes induced by theta-burst stimulation (TBS) during the third week of age but not later. We also demonstrate that the direction of synaptic modification (LTD or LTP) is related to the level of synergy involving different types of 5-HT receptors. Finally, we show that this 5-HT-dependent plasticity is absent in animals whose postnatal development is influenced by exposure to a model of chronic stress that disrupt the serotonergic system^35^.

## Materials and methods

### Animals

All procedures were approved by the Institutional Animal Care Committee of the Institute of Biomedical Sciences (ICB), University of São Paulo, Brazil (CEUA ICB/USP # 1198240718, of July 25, 2018). All methods were carried out in accordance with relevant guidelines and regulations and are reported in accordance with ARRIVE guidelines. Wistar rats of either sex at the age of postnatal day 14–16 (P14-16), or 24–26 (P24-26) were used. Rats were kept in the animal facility of the Department of Physiology and Biophysics at ICB/USP under standard conditions: 23 ± 2 °C, 12 h light/dark cycle, lights on 6h00 a.m., food and water ad libitum, cages measuring 40 × 33 × 17 cm (in width, length and height respectively), lighting ~ 200 lx. Some animals underwent to a Maternal Separation protocol (MS), so all rats were allocated into four experimental groups: P14-16 non-separated rats (control), P14-16 MS rats, P24-26 non-separated rats (control), P24-26 MS rats. The P14-16 has been chosen as the early life temporal window considering that the PFC is an associative brain structure and its postnatal development is subordinated to the maturation of sensory areas^36,37^. At P14 somatosensory and auditory cortex have completed their principal connectivity and layer organization. From 10 days onwards, rodents open their eyes and vision becomes an important sensory channel. Thus, P14 corresponds to an age when the brain is still under development, but with most sensory systems already structured and able to provide complex stimuli for the PFC.

### Maternal separation

Nulliparous female Wistar young rats were mated (one male and two females). Male animals were removed from the cage as soon as the females became pregnant. Litters were reduced to 8 pups on postnatal day 2 (P2), maintaining an equal number of males and females. From P2 onwards, the dams were separated from the pups for 3 h daily, until P14. During separation, pups were kept in their nest in the original cages without any heating source, while the mothers were placed in separate cages in the same room. We always used the same cages during the protocol. At the end of the 3 h of separation, the mothers were returned to their original cages, along with their respective litters. Non-separated animals were handled only during the days of cage maintenance (twice a week) and did not undergo any additional handling/intervention.

### Preparation and maintenance of brain slices

Animals were deeply anesthetized through isoflurane inhalation (5% isoflurane in oxygen) and then decapitated. The brain was quickly removed, glued to an iron platform, and submerged in cooled (0 °C) oxygenated (5% CO_2_–95% O_2_) dissection buffer, containing the following (in mM): 206 sucrose, 25 NaHCO_3_, 2.5 KCl, 10 MgSO_4_, 1.25 NaH_2_PO_4_, 0.5 CaCl_2_, and 11 d-glucose). The brain was sectioned by a vibratome and coronal cortical slices (300–350 μm) containing the prefrontal cortex (PFC) were obtained. Slices were transferred to a holding chamber containing artificial CSF (ACSF), with the following (in mM): 126 NaCl, 26 NaHCO3, 2.5 KCl, 1.45 NaH2PO4, 1 MgCl2, 2 CaCl2, and 9 d-Glucose. Slices were kept oxygenated at room temperature (RT, 20–25 °C) for at least 1 h before electrophysiological experiments.

### Electrophysiological recordings and electrical stimulation

Coronal cortical slices were transferred in a submersion-type recording chamber upon a modified microscope stage. Slices were constantly perfused with oxygenated ACSF (5% CO_2_–95% O_2_) and maintained at RT throughout the experiments. Whole-cell patch-clamp recordings were made in neurons located in layer 5 of the prelimbic region of PFC. Recordings were performed from regular spiking pyramidal-shaped cells. Recording electrodes pipettes were fabricated from borosilicate glass (Garner Glass; California, USA) with input resistances of ∼4–6 MΩ and were filled with intracellular solution containing the following (in mM): 117 K-gluconate, 10 HEPES, 2 Na_2_ATP, 0.4 Na_3_GTP, 1 MgCl_2_, 0.1 EGTA, 13 KCl, 0.07 CaCl_2_, 0.1–0.5% biocytin, pH 7.3, and osmolarity 290 mOsm. We used a visualized slice setup under a differential interference contrast-equipped microscope to perform our electrophysiology experiments. Postsynaptic responses were recorded in current-clamp mode by using a Multiclamp 700B amplifier and pClamp software (Molecular Devices; California, USA). Cells were recorded in current clamp mode at free membrane potential (I = 0). We included in our data only recordings from cells with access resistance < 20 MΩ, and input resistance were > 100 MΩ and < 1000 MΩ during the whole recording period. Data were discarded if any of these values changed > 20% during the baseline recording. We included in our data only recordings from cells with membrane potentials <  − 65 mV during the baseline recording after correction for liquid junction potential (3.378 mV). For all experimental groups, no more than two cells were recorded from the same animal.

Evoked postsynaptic potentials (EPSPs) were recorded by electrically stimulating inputs to pyramidal neurons of layer 5 (0.1 Hz; 0.2 ms). A concentric bipolar electrode (125 μm diameter) was placed on superficial layer 1 to deliver stimulation and activate synapses of pyramidal neurons whose cellular body is located in layer 5. The intensity of stimulation was increased gradually by steps of 5 μA from a subthreshold level until a response was evoked. The intensity was then adjusted to evoke 2–5 mV responses.

Changes in synaptic strength caused by 5-HT or 5-HT_1A/2A/7_ receptors agonists were quantified as changes in the initial amplitude of the postsynaptic potential normalized by the mean baseline response obtained during the first 10 min of stable recordings. In experiments aimed to assess the action of 5-HT and 5-HT_1A/2A/7_ receptors agonists on synaptic transmission, the amplitude of the EPSPs was normalized to the averaged EPSPs value of the baseline (the 10 min period before the drug).

A theta-burst stimulation (TBS) protocol was used to induce synaptic plasticity. The TBS consisted of 3 repeated trains of 13 bursts, being 60 s the interval between each train. The frequency of bursts for each train was 5 Hz (200 ms interburst interval). Each burst contained a four-pulse train with a frequency of 100 Hz. Changes in synaptic response were normalized to the baseline recorded during the first 10 min of stable recording, and whole-cell recording was maintained for 40 min after baseline. This protocol has shown efficacy in inducing plasticity in the PFC in experiments conducted under our same conditions^38^.

In some experiments, we used the paired-pulse ratio (PPR) to determine the pre- or postsynaptic nature of changes induced by 5-HT receptors activation and/or TBS. PPR values were obtained by applying 4 four-pulse trains at 10 Hz. The PPR applied before the baseline was compared with the PPR observed by the end of the experiment.

Changes in membrane potential or input resistance caused by 5-HT or 5-HT_1A/2A/7_ receptors agonists were quantified as changes in the initial value normalized by the mean baseline response obtained during the first 10 min of stable recordings. In experiments aimed to assess the action of 5-HT and 5-HT_1A/2A/7_ receptors agonists on synaptic transmission, the amplitude of the EPSPs was normalized to the averaged EPSPs value of the baseline (the 10 min period before the drug).

### Pharmacology

To assess the effects of 5-HT on synaptic transmission and synaptic plasticity, 5-HT (Serotonin, 50 μM), a 5-HT_2A_ antagonist (Ketanserin, 10 µM), a 5-HT_1A_ antagonist (WAY-100635, 1 µM), a 5-HT_7_ antagonist (SB-269970, 10 µM), a 5-HT_2A_ agonist (TCB-2, 10 µM), a 5-HT_1A_ agonist (8-OH-DPAT, 1 µM) and a 5-HT_7_ agonist (LP-44, 2 µM) were used in our electrophysiological experiments. In some cases, 5-HT, or one 5-HT receptor agonist were added in the bath. Application occurred through the exchange of recording solution in the perfusion. In some cases, tissues were placed in an ACSF solution containing 5-HT and/or one 5-HT receptor antagonist throughout the whole experiment. Stock solutions for all drugs were prepared with deionized water. The final concentration was calculated based on the volume of the recording chamber, tubes, and small ACSF container. After addition, the drug remained in the perfusion solution until the end of the cellular recording.

### Forced Swim Test

The Forced Swim Test (FST) was performed in P24-26 non-separated (control) and MS animals. The test consisted of a 15 min training session, followed by a 5 min test session on the next day, with the sessions being filmed for further analysis. The test was conducted in a cylindrical tank (120 cm in height, 25 cm in diameter) with water at 24 ± 1 °C and 60 cm level. The FST analysis parameter consisted of the immobility time (when 3 or more paws of the animal were immobile) during the test session.

### Data analysis

Electrical features under current injection in pyramidal neurons from layer 5 were extracted using the Electrophys Feature Extraction Library (eFEL) [https://github.com/BlueBrain/eFEL]. The steady state voltage was measured using the last 10% of the stimulus duration. For each current step the spike times were saved and the interspike intervals were calculated.

Synaptic change induced by 5-HT, TCB-2, 8-OH-DPAT or LP-44 was quantified by calculating the normalized EPSPs amplitude average of the last 5 min of recording (25–30 min) and by comparing this value with the normalized EPSPs average of the last 5 min of the baseline (0–10 min). In experiments where 5-HT was applied under the presence of 5-HT_1A/2A/7_ receptor antagonists and after a baseline (0–10 min), the effect of two periods was considered for the analysis (15–20 min and 25–30 min).

Synaptic change induced by TBS was quantified by calculating the normalized EPSPs amplitude average of the last 5 min (35–40 min) and by comparing this value with the normalized EPSPs average of the last 5 min of the baseline (0–10 min).

In PPR experiments, post-synaptic response was normalized to the EPSP of the first pulse. Data from 5 train-repetitions were averaged to calculate the PPR and the value relative to the fourth pulse was considered for analysis. The PPR of the baseline was compared with the PPR recorded at the end of the experiment.

Changes of input resistance and membrane potential induced by 5-HT_1A/2A/7_ receptor agonists were quantified by calculating the normalized average values of the last 5 min (25–30 min) and by comparing these values with the normalized average values of the last 5 min of the baseline (the first 10 min of stable recordings).

In experiments with animals subjected to MS, the effect due to litter differences was minimized by distributing the animals of a given litter as much as possible among the experimental groups. In this way, all experimental groups of MS animals were made of pups obtained from all the MS procedures performed. The FST data analysis was made considering the percentage time of immobility in respect to the full time of the experiment (5 min).

### Statistical methods

For all data, normality was tested by means of the Shapiro–Wilk test. In experiments where data were normally distributed, statistical analysis was performed by using parametric tests. When the mean differences were affected by one variable, with the experimental groups being two independent or two paired samples, the unpaired t-test or the paired t-test was used respectively. When one variable was studied and the experimental groups were three paired samples, we adopted the two-way repeated measures ANOVA and the LSD Fisher post-hoc test was used for comparisons. When one variable was studied and the experimental groups were more than two independent samples, we adopted the one-way ANOVA and the Tukey HSD post-hoc test was used for comparisons. Finally, when the mean differences between groups were affected by two independent variables, the two-way ANOVA was used.

In experiments where data were not normally distributed and the mean differences between groups were split on one variable, statistical analysis was performed by using non-parametric tests. When the experimental groups where two independent or two paired samples, the Mann–Whitney U test or the Wilcoxon signed rank test was used respectively. When groups were more than two independent samples, the Kruskal Wallis test was adopted and the Dunn's post-hoc test was used for comparisons. In experiments where data were not normally distributed and the mean differences between groups was split on two variables, data were first log10 transformed for achieving normality. Then, statistical analysis was performed by using the two-way ANOVA.

In the analysis where the distribution of plasticity events was studied, the specific outcome (LTP, LTD, No plasticity) related to each experiment was assessed by means of the Wilcoxon signed-rank test. For each cell, the EPSP mean values of the baseline where compared with the EPSP mean values of the last 10 minutes of the post induction period. Those cells that showed no statistical difference (p > 0.05 on the Wilcoxon signed-rank test) were classified as “no plasticity”. Those cells that showed statistical difference (p < 0.05 on the Wilcoxon signed-rank test) were classified as LTP (if the mean of the normalized EPSP values of the last 10 minutes of the post induction period was higher than 100%) or LTD (if the mean of the normalized EPSP values of the last 10 minutes of the post induction period was lower than 100%). Cells Statistical differences in the distribution of plasticity outcomes between experimental groups was calculated by using the Chi-square test. Statistical differences in the distribution of plasticity outcomes between experimental groups was calculated by using the Chi-square test.

All experimental groups were considered significantly different for p values lower than 0.05.

## Results

### Spiking pattern of layer V neurons in P14-16 animals

**Figure 1:**
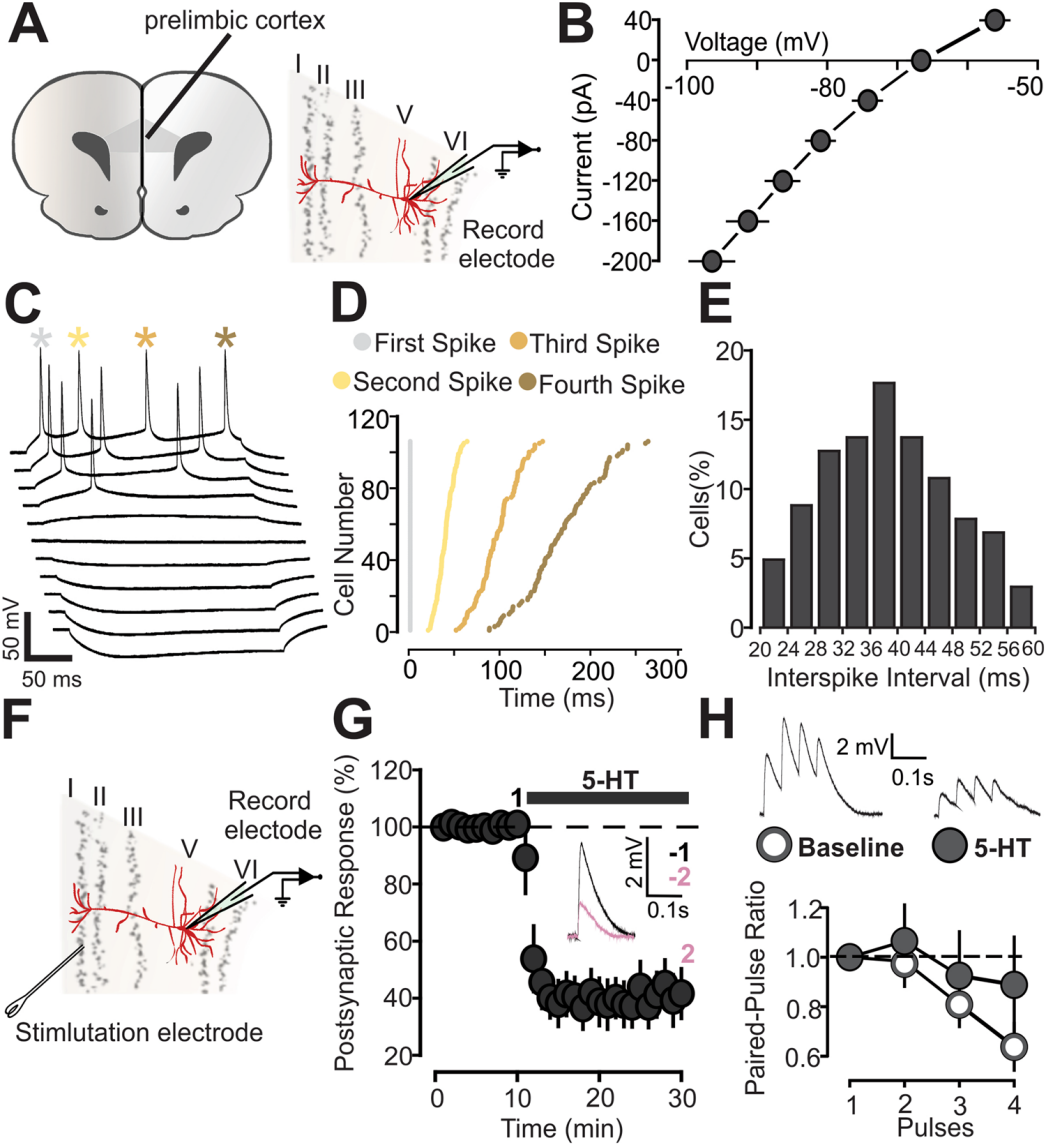
5-HT reduces the amplitude of postsynaptic responses. (**A**) Scheme showing the prelimbic region of medial PFC on a coronal slice and the position of the stimulation electrode and recorded neurons. (**B**) Current–voltage (I–V) plots of PFC layer 5 neurons at P14-16. (**C**) Example of action potentials obtained by current injection in pyramidal neurons from layer 5. (**D**) The graph shows the cumulative relative spike intervals of neurons recorded from layer 5 at P14-16. The interval values were normalized with respect to the first spike time (t = 0). (**E**) The graph shows the frequency histogram for the interspike intervals of neurons recorded from layer 5 at P14-16. (**F**) Scheme showing the position of the stimulation electrode and recorded neurons in the prelimbic region of medial PFC. (**G**) The graph shows the EPSPs recorded in P14-16 animals before and during 5-HT (50 µM) bath application. The EPSPs were normalized to the mean of responses recorded during the baseline. The black bar represents the period of application of 5-HT in the bath. The EPSPs were normalized to the mean of responses recorded during the baseline and the bars represent the SEM. Traces show the results of a representative experiment the average of postsynaptic responses recorded before (1: 5 to 10 min, black line) and after (2: 25 to 30 min, magenta line) application of 5-HT. (**H**) Graph showing the paired pulse ratio of EPSPs in P14-16 animals. Two different conditions are compared: control (white inserts) and 5-HT application (gray inserts). Postsynaptic responses were normalized to the response caused by the first pulse. The EPSPs were normalized to the mean of responses recorded during the baseline and the bars are respect to the SEM. Traces show the results of a representative experiment with the average of postsynaptic responses recorded before the baseline and 20 min after the bath application of 5-HT.

Initially, before investigating the effects of 5-HT on synaptic transmission and plasticity, we recorded and studied the spiking properties of PFC layer 5 neurons (Fig. 1A) in the early postnatal period (P14-16), in order to find out whether they constitute a homogeneous population in terms of biophysical properties. The current–voltage (I–V) plots for a group of recorded neurons show little variability (Fig. 1B: n = 11). All recorded neurons were consistent with a regular spiking firing pattern (Fig. 1C; Fig. 1D: n = 104). All cells begin firing at a short latency following onset of the depolarizing current step and then accommodate. Figure 1E shows the frequency histogram for the interspike intervals obtained from the recorded neurons. The values are normally distributed, suggesting that the population is relatively homogeneous and not organized in separated clusters (Fig. 1E: n = 104; p = 0.125 based on the Shapiro Wilk Test).

### 5-HT modulation of synaptic transmission in P14-16 animals

We then investigated the direct effects of 5-HT on EPSPs in P14-16 animals, regardless of the induction of activity-dependent plasticity. As shown in Fig. 1F, we stimulated superficial layers to evoke the post-synaptic response in layer 5 cells. Application of 5-HT (50 µM) in the bath produced a 60% reduction of EPSPs (Fig. 1G: 5-HT. 10 cells, 45.8 ± 8.2, p = 5 × 10^–5^, based on a paired t-test; Supplementary Figure A: 5-HT. 10 cells, slope, 43.4 ± 11.1, p = 6.14 × 10^–5^, on a paired t-test). To evaluate the pre- or postsynaptic nature of this phenomenon, we recorded the EPSPs ratios caused by a train of 4 paired pulses (10 Hz), comparing two situations: before and after the application of 5-HT (Fig. 1H: 9 cells; baseline 0.64 ± 0.09; 5-HT, 0.89 ± 0.19). No statistical differences in EPSPs were found between the two conditions (p = 0.692, based on a paired t-test). This suggests that 5-HT decreases the postsynaptic response by acting postsynaptically.

Subsequently, we investigated the serotonergic receptors involved in the 5-HT-induced depression. We used antagonists and agonists for 5-HT_1A_, 5-HT_2A,_ and 5-HT_7_ receptors, the main 5-HT receptors modulating excitability in PFC neurons during the early postnatal period^29^.

**Figure 2:**
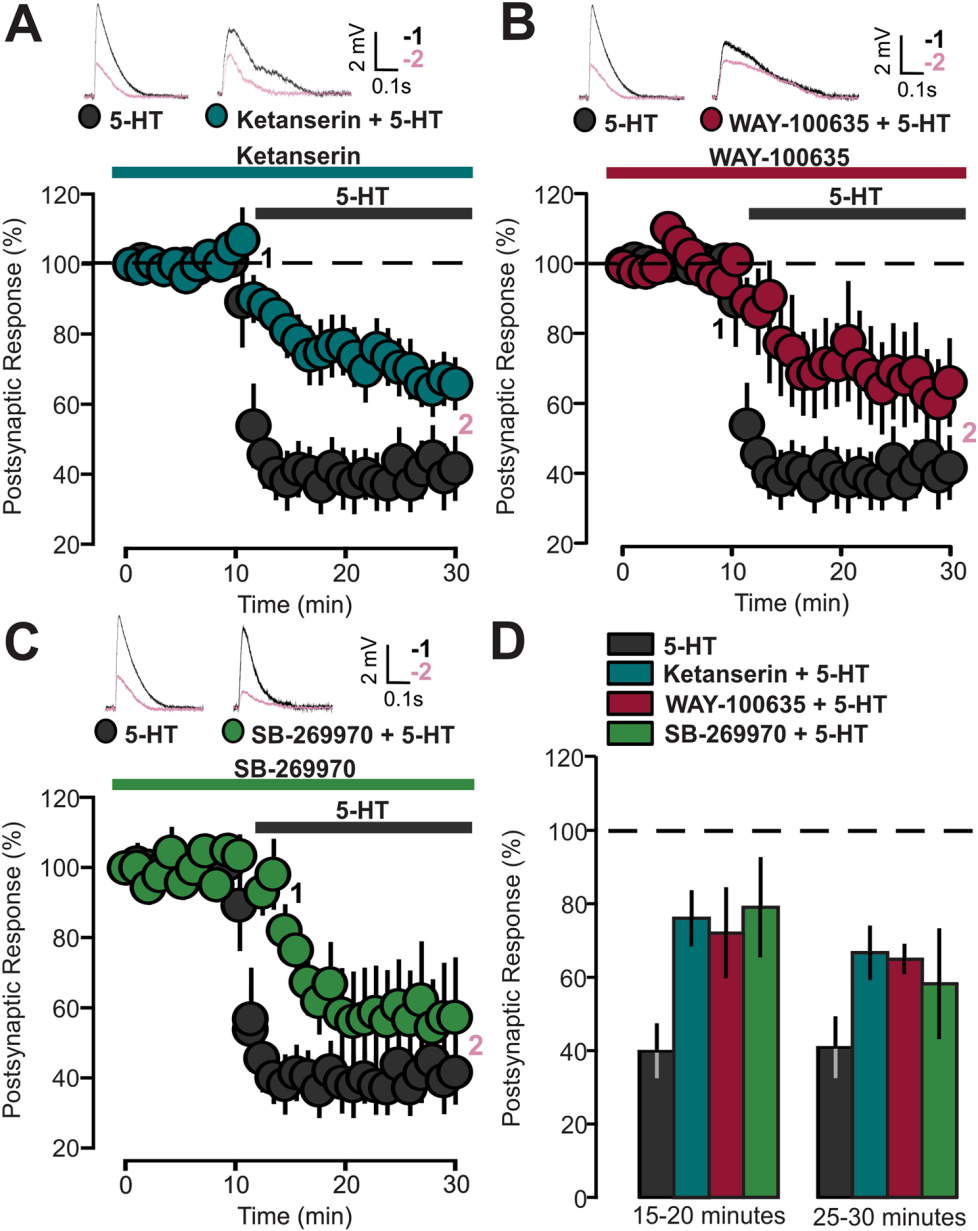
5-HT_2A_, 5-HT_1A_, and 5-HT_7_ receptors antagonists inhibit the effects of 5-HT on synaptic transmission. (**A**) The graph shows the EPSPs recorded in P14-16 animals before and during 5-HT (50 µM) bath application, in the presence or not of the 5-HT_2A_ receptor antagonist. Two different conditions are compared: 5-HT (dark grey inserts, 5-HT, 50 µM) and 5-HT_2A_ receptor antagonist + 5-HT (blue inserts; Ketanserin, 10 µM, + 5-HT, 50 µM). The EPSPs were normalized to the mean of responses recorded during the baseline and the bars represent the SEM. Traces show the results of a representative experiment with the average of postsynaptic responses recorded before (1: 5 to 10 min, black line) and after (2: 25 to 30 min, magenta line) application of 5-HT. (**B**) The graph shows the EPSPs recorded in P14-16 animals before and after 5-HT (50 µM) application in the presence or not of 5-HT_1A_ receptor antagonist. Two different conditions are compared: 5-HT (dark grey inserts, 5-HT) and 5-HT_1A_ receptor antagonist + 5-HT (red inserts, WAY-100635, 1 µM, + 5-HT, 50 µM). The EPSPs were normalized to the mean of responses recorded during the baseline and the bars indicate the SEM. Traces show the results of a representative experiment with the average of postsynaptic responses recorded before (1: 5 to 10 min, black line) and after (2: 25 to 30 min, magenta line) application of 5-HT. (**C**) The graph shows the EPSPs recorded in P14-16 animals before and after 5-HT (50 µM) application, with two different conditions being compared: 5-HT (dark gray inserts, 5-HT) and 5-HT_7_ receptor antagonist + 5-HT (green inserts, SB-269970, 10 µM, + 5-HT, 50 µM). The EPSPs were normalized to the mean of responses recorded during the baseline and the bars represent the SEM. Traces show the results of a representative experiment with the average of postsynaptic responses recorded before (1: 5 to 10 min, black line) and after (2: 25 to 30 min, magenta line) application of 5-HT. (**D**) Summary of the depressive effects of 5-HT. The graph bars show the EPSP values recorded during two different periods of the experiment: 15–20 min and 25–30 min.

Specific antagonists for 5-HT_1A_ (WAY-100635, 1 µM), 5-HT_2A_ (Ketanserin, 10 µM) and 5-HT_7_ (SB-269970, 10 µM) receptors were used in the following experiments. In the presence of WAY-100635, Ketanserin, or SB-269970, bath application of 5-HT resulted in a significant (30–40%) reduction of the EPSPs (Fig. 2A: Ketanserin + 5-HT, 13 cells, 66.7 ± 6.8, p = 0.0003, based on a paired t-test; Fig. 2B: WAY-100635 + 5-HT, 8 cells, 58.8 ± 13.3 SEM, p = 0.032, based on a paired t-test; Fig. 2C: SB-269970 + 5-HT, 8 cells, 62 ± 17.1, p = 0.024, based on a paired t-test). The averages of the EPSPs amplitude recorded during two periods of 5-HT application, 15–20 min and 25–30 min, are shown in Fig. 2D. WAY-100635 and Ketanserin significantly reduced the depressive action of 5-HT (Fig. 2D: p = 0.007, on a two-way repeated measure ANOVA, p = 0.007 for the 5-HT versus Ketanserin and p = 0.048 for the 5-HT versus WAY-100635 comparisons on an LSD Fisher test).

**Figure 3:**
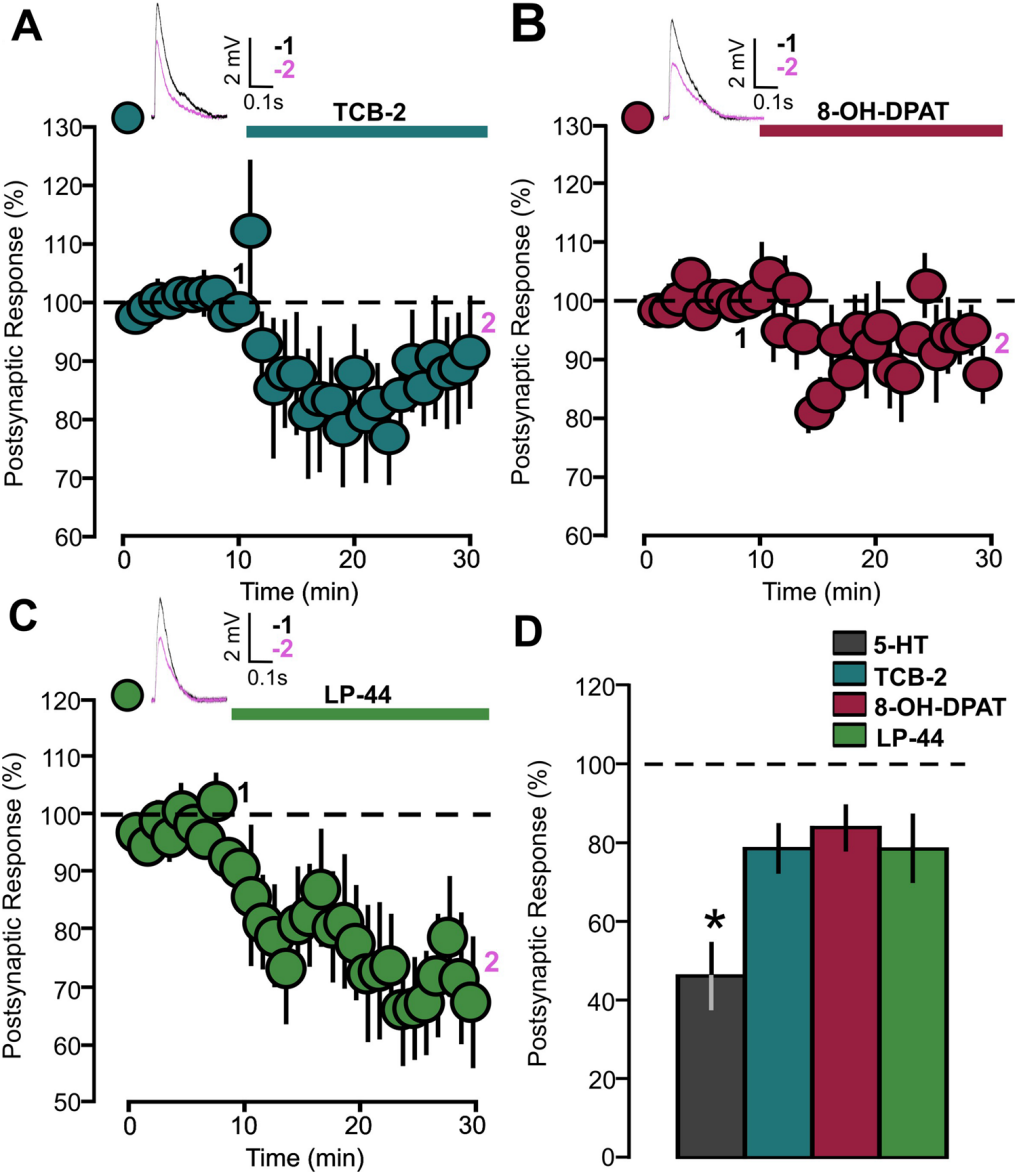
5-HT_1A_, 5-HT_2A_ and 5-HT_7_ receptors agonists reduce the amplitude of synaptic responses. (**A**) The graph shows the EPSPs recorded in P14-16 animals before and during 5-HT_2A_ receptor agonist TCB-2 (10 µM) bath application. The EPSPs were normalized to the mean of responses recorded during the baseline. Traces show the results of a representative experiment with the average of postsynaptic responses recorded before (1: 5 to 10 min, black line) and after (2: 25 to 30 min, magenta line) application of TCB-2. (**B**) The graph shows the EPSPs recorded in P14-16 animals before and during 5-HT_1A_ receptor agonist 8-OH-DPAT (1 µM) bath application. The EPSPs were normalized to the mean of responses recorded during the baseline. Traces show the results of a representative experiment with the average of postsynaptic responses recorded before (1: 5 to 10 min, black line) and after (2: 25 to 30 min, magenta line) application of 8-OH-DPAT. (**C**) The graph shows the EPSPs recorded in P14-16 animals before and during 5-HT_7_ receptor agonist LP-44 (2 µM) bath application. The EPSPs were normalized to the mean of responses recorded during the baseline. Traces show the results of a representative experiment with the average of postsynaptic responses recorded before (1: 5 to 10 min, black line) and after (2: 25 to 30 min, magenta line) application of LP-44. (**D**) Graph showing the effects of 5-HT and 5-HT_1A/2A/7_ agonists. The averaged synaptic responses recorded during the 25–30 min period are reported. EPSPs were normalized to the mean of responses recorded during the baseline.

TCB-2 (10 μM), 8-OH-DPAT (1 μM) and LP-44 (2 μM) were employed for selective 5-HT_2A_, 5- HT_1A_ and 5-HT_7_ receptor activation, respectively. The agonist was applied after 10 min of baseline recording. We found that each of the three agonists reduced EPSPs and this effect is statistically significant (Fig. 3A: TCB-2, 12 cells, 78.6 ± 6.4, p = 0.034, based on a paired t-test; Fig. 3B: 8-OH-DPAT, 14 cells, 83.9 ± 6.2, p = 0.027, based on a paired t-test; Fig. 3C: LP-44, 10 cells, 78.5 ± 7.8, p = 0.046, based on a paired t-test).

Figure 3D summarizes our results obtained from experiments carried out with 5-HT and 5-HT_1A/2A/7_ agonists. Synaptic depression caused by 5-HT is stronger when compared to the effect induced by bath application of each one of the three agonists (Fig. 3D: p = 0.0003, on a one-way ANOVA; p = 0.003 for the 5-HT versus TCB-2 comparison, p = 0.001 for the 5-HT versus 8-OH-DPAT comparison and p = 0.005 for the 5-HT versus LP-44 comparison, on a Tukey HSD test).

### 5-HT modulation of synaptic plasticity in P14-16 animals

**Figure 4:**
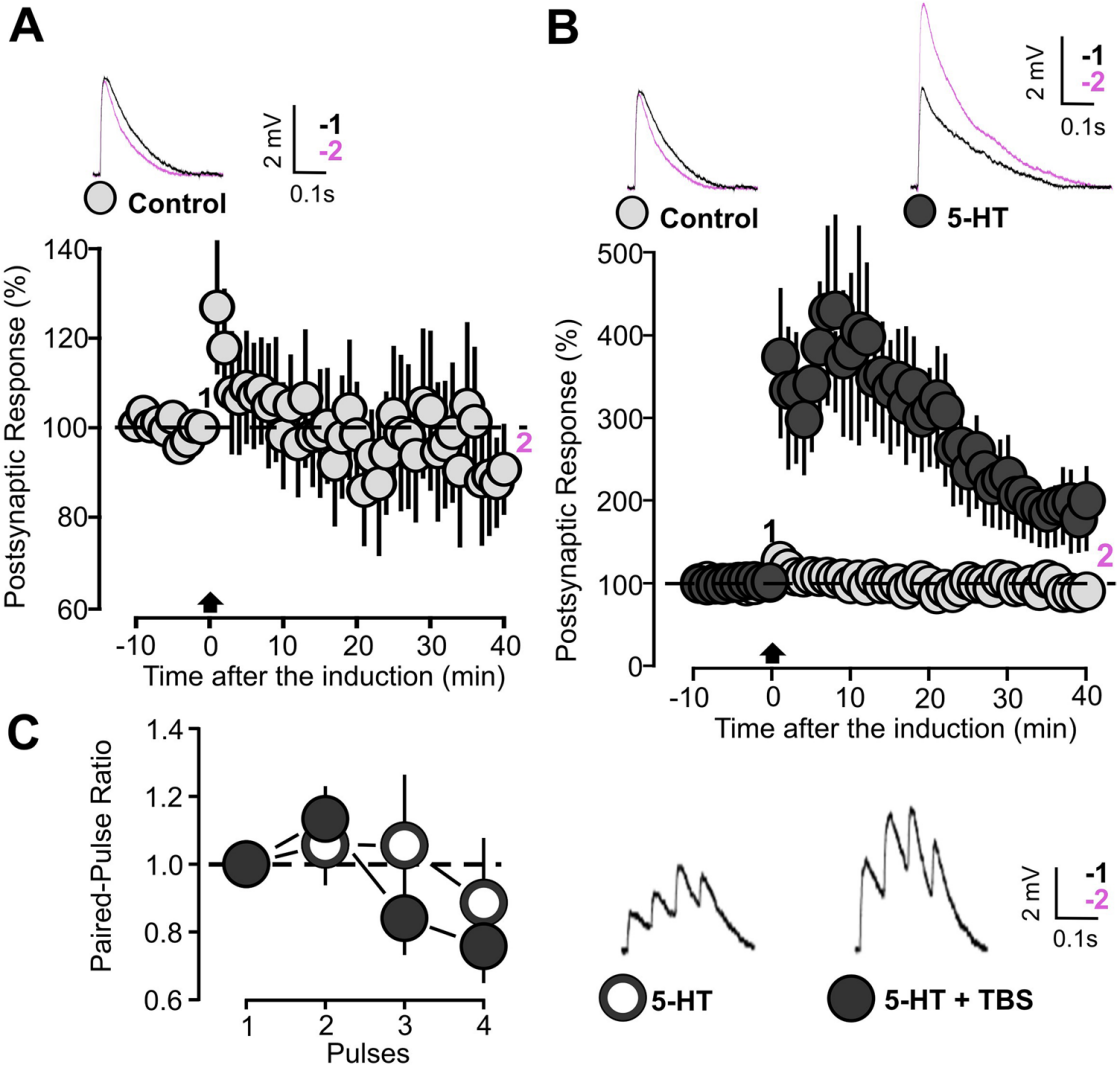
TBS induction causes LTP in P14-16 animals slices under the modulation of 5-HT. (**A**) The graph shows the EPSPs recorded in P14-16 animals before and after TBS induction. The EPSPs were normalized to the mean of responses recorded during the baseline. Traces show the average of postsynaptic responses recorded before (1: baseline, − 5 to 0 min, black line) and after the TBS induction (2: post induction, 35–40 min, black line). (**B**) The graph shows the EPSPs recorded in P14-16 animals before and after TBS induction in the presence or not of 5-HT. The EPSPs were normalized to the mean of responses recorded during the baseline. Two different conditions are compared: control (white insert) and 5-HT (gray inserts, 50 µM). Traces show the results of a representative experiment with the average of postsynaptic responses recorded before (1: baseline, − 5 to 0 min, black line) and after the TBS induction (2: post induction, 35–40 min, black line). (**C**) Graph showing the paired pulse ratio of 4 pulses (10 Hz) EPSPs in slices treated with 5-HT (50 µM). Two different conditions are compared: before (white inserts) and after TBS induction (gray inserts). Postsynaptic responses were normalized to the response caused by the first pulse. Traces show the results of a representative experiment with the average of postsynaptic responses recorded before the baseline and 40 min after the TBS induction.

We used the TBS protocol to study how 5-HT modulates activity-dependent synaptic plasticity in P14-16 animals. In slices not treated with 5-HT, the TBS induction did not cause significant changes in the EPSP amplitude (Fig. 4A: 10 cells, 91.4 ± 12.2, p = 0.532 on a paired t-test). TBS was then used in slices treated with the neuromodulator throughout the whole recording period. Under these new conditions, we observed a substantial increase in postsynaptic responses by 100% (Fig. 4B: control: 10 cells, 91.4 ± 12.2, p = 0.532, on a paired t-test; 5-HT: 15 cells, 218.6 ± 40.1, p = 0.015 on a Wilcoxon signed rank test; Supplementary Figure B: slope; Control: 10 cells, 112.15 ± 15.6, p = 0.364, based on a paired t-test; 5-HT: 15 cells, 274.2 ± 78.6, p = 0.005 based on a Wilcoxon signed rank test). To evaluate the possibility of presynaptic changes, four paired pulses were delivered at the beginning and at the end of the experiments. The PPRs obtained in these two situations were compared in Fig. 4C and no statistical differences were found (Fig. 4C: 13 cells, baseline, 0.89 ± 0.18; post TBS, 0.76 ± 0.10; p = 0.340, based on a paired t-test).

### Inhibition of 5-HT dependent plasticity in P14-16 animals by 5-HT_1A/2A/7_ antagonists

**Figure 5:**
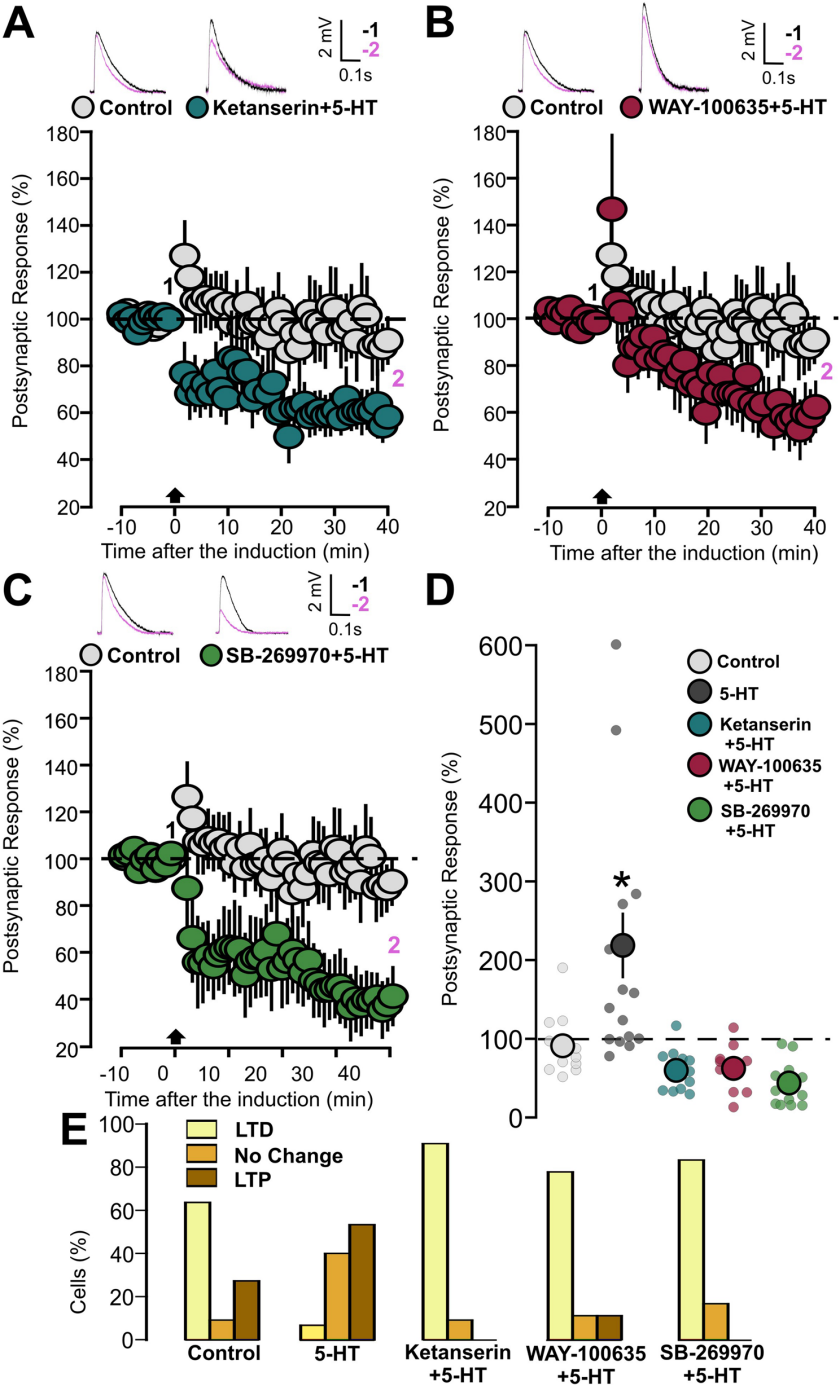
5-HT modulation inhibited by one of the 5-HT_1A/2A/7_ receptors antagonists shift synaptic plasticity from LTP to LTD. (**A**) The graph shows the EPSPs recorded in P14-16 animals before and after TBS induction. The EPSPs were normalized to the mean of responses recorded during the baseline. Two different conditions are compared: control (light grey inserts) and 5-HT (50 µM) together with the 5-HT_2A_ receptor antagonist (blue inserts, Ketanserin, 10 µM). Traces show the results of a representative experiment with the average of postsynaptic responses recorded before (1: baseline, − 5 to 0 min, black line) and after the TBS induction (2: post induction, 35–40 min, black line). (**B**) The graph shows the EPSPs recorded in P14-16 animals before and after TBS induction. The EPSPs were normalized to the mean of responses recorded during the baseline. Two different conditions are compared: control (light grey inserts) and 5-HT (50 µM) together with the 5-HT_1A_ receptor antagonist (red inserts, WAY-100635, 1 µM). Traces show the results of a representative experiment with the average of postsynaptic responses recorded before (1: baseline, − 5 to 0 min, black line) and after the TBS induction (2: post induction, 35–40 min, black line). (**C**) The graph shows the EPSPs recorded in P14-16 animals before and after TBS induction. The EPSPs were normalized to the mean of responses recorded during the baseline. Two different conditions are compared: control (light grey inserts, 50 µM) and 5-HT together with the 5-HT_7_ receptor antagonist (green inserts, SB-269970, 10 µM). Traces show the results of a representative experiment with the average of postsynaptic responses recorded before (1: baseline, − 5 to 0 min, black line) and after the TBS induction (2: post induction, 35–40 min, black line). (**D**) Scatter graph showing the EPSPs recorded during the 35–40 min period after TBS induction, for individual experiments (small dots) and for the mean values of the experimental group (large dots, with bars representing the SEM). Five experimental groups are compared based on the condition of drug incubation (Control, 5-HT, Ketanserin + 5-HT, WAY-100635 + 5-HT and SB-269970 + 5-HT). EPSPs values were normalized to the mean of responses recorded during the baseline. (**E**) The graph shows the percentage of cells exhibiting LTP, LTD or no change in five experimental groups (Control, 5-HT, Ketanserin + 5-HT, WAY-100635 + 5-HT and SB-269970 + 5-HT).

We then investigated the involvement of 5-HT receptors in the modulation of TBS-induced plasticity by means of 5-HT_1A/2A/7_ antagonists. The TBS was delivered in slices that were treated with 5-HT, along with one specific 5-HT receptor antagonist. When TBS was applied in the presence of 5-HT and 5-HT_2A_ receptor antagonist (Ketanserin, 10 µM), a reduction of synaptic response was observed (Fig. 5A: 11 cells, 59.82 ± 8.2, p = 0.0009, based on a paired t-test). Similarly, when the same induction protocol was applied to tissues treated with 5-HT and 5-HT_1A_ receptor antagonist (WAY-100635, 1 µM), we found that the EPSPs were significantly decreased (Fig. 5B: 11 cells, 62.2 ± 10.6, p = 0.006, based on a paired t-test). Finally, a third group of slices was subjected to the modulation of 5-HT hindered by the 5-HT_7_ receptor antagonist (SB-269970, 10 µM). The TBS induction also in this case resulted in LTD (Fig. 5C: 12 cells, 43.7 ± 8.2, p = 0.002, based on a Wilcoxon signed rank test).

Figure 5D summarizes the effect of TBS and 5-HT under the influence of 5-HT_1A/2A/7_ antagonists. 5-HT coupled with TBS leads to LTP while pharmacological inhibition of either 5-HT_2A,_ 5-HT_1A_ or 5-HT_7_ receptor activity results in LTD expression. Post induction synaptic responses recorded from cells treated with 5-HT are significantly higher when compared to the other groups (Fig. 5D: p = 2 × 10^–6^ based on a Kruskal Wallis test; p = 0.026 for 5-HT versus Control; p = 0.0002 for 5-HT versus 5-HT + Ketanserin; p = 0.0004 for 5-HT versus 5-HT + WAY-100635; p = 1.9 × 10^–7^ for 5-HT versus 5-HT + SB-269970. All comparisons were done on a Dunn post hoc test).

The facilitating effect of 5-HT receptors on TBS induced synaptic plasticity may result from modifications in the distribution of the probability of the three possible induction outcomes: LTP, LTD or no plasticity. Otherwise, 5-HT may act by increasing the quantitative impact of the main form of plasticity being expressed, leaving the general probability distribution unchanged. In order to study what kind of alteration is induced by 5-HT in this sense, we have carried out a study of the plasticity outcome distribution in the 5 experimental groups (Control, 5-HT, 5-HT + Ketanserin, 5-HT + WAY-100635 and 5-HT + SB-269970). The result of this analysis is shown in Fig. 5E. 5-HT reduces the number of cells that undergo LTD and increases those that exhibit LTP. This effect is reversed by the presence of one of the three 5-HT antagonists (Fig. 5E: p = 0.0003, based on a Chi squared test). This analysis suggests that 5-HT favors LTP in TBS-dependent plasticity by increasing the probability that the induction results in a potentiation event.

**Figure 6:**
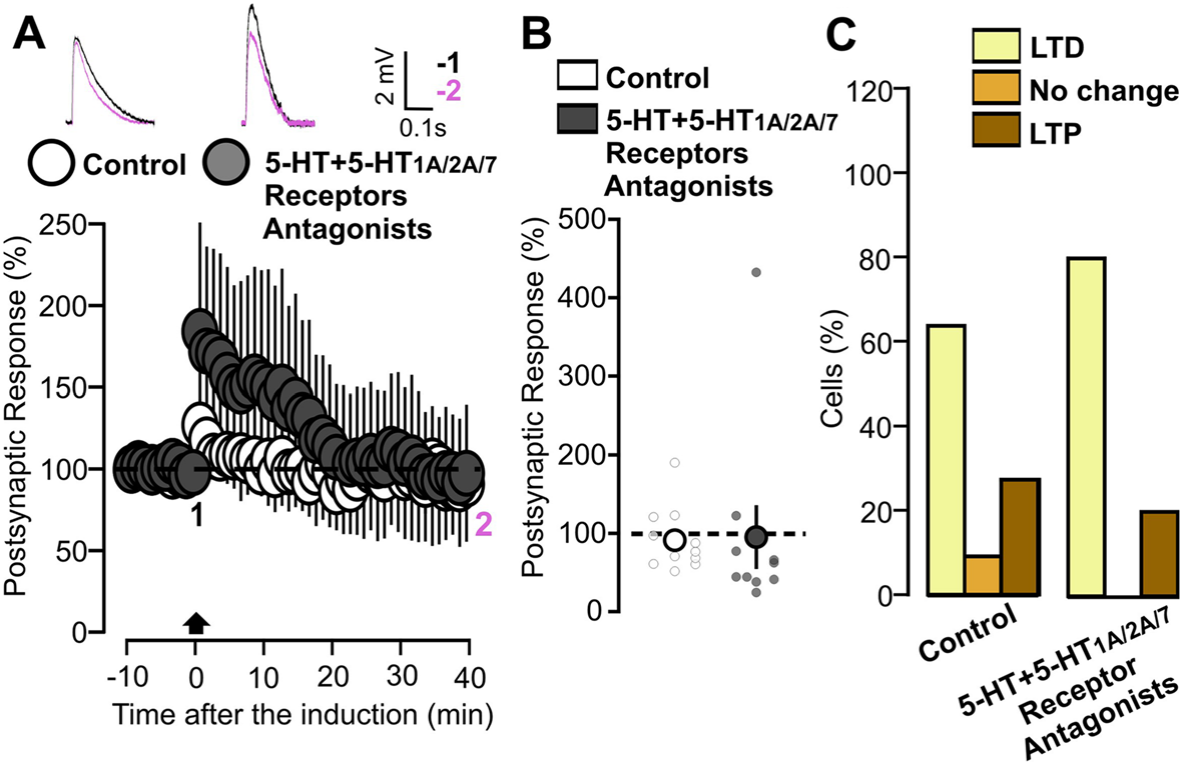
Application of 5-HT together with the three 5-HT_1A/2A/7_ receptors antagonists has no effect on TBS synaptic plasticity. (**A**) The graph shows the EPSPs recorded in P14-16 animals before and after TBS induction. The EPSPs were normalized to the mean of responses recorded during the baseline. Two different conditions are compared: control (white inserts) and 5-HT together with the three 5-HT_1A/2A/7_ receptor antagonists (dark grey inserts: 5-HT, 50 µM, Ketanserin, 10 µM, WAY-100635, 1 µM, SB-269970, 10 µM). Traces show the results of a representative experiment with the average of postsynaptic responses recorded before (1: baseline, − 5 to 0 min, black line) and after the TBS induction (2: post induction, 35–40 min, black line). (**B**) Scatter graph showing the averaged EPSPs recorded during the 35–40 min period after TBS induction, for individual experiments (small dots) and for the mean values of the experimental group (large dots, with bars representing the SEM). Two experimental groups are compared based on the condition of drug incubation: control and 5-HT + 5-HT_1A/2A/7_ receptors antagonists (5-HT, 50 µM, Ketanserin, 10 µM, WAY-100635, 1 µM, SB-269970, 10 µM). EPSPs values were normalized to the mean of responses recorded during the baseline. (**C**) The graph shows the percentage of cells exhibiting LTP, LTD or no change in two experimental groups: control and 5-HT + 5-HT_1A/2A/7_ receptors antagonists (5-HT, 50 µM, Ketanserin, 10 µM, WAY-100635, 1 µM, SB-269970, 10 µM).

Subsequently, we studied the effect of the 5-HT_1A/2A/7_ antagonists on 5-HT dependent plasticity, applying them together in the bath along with 5-HT during the experiments. When the cells were stimulated by TBS in the presence of 5-HT and the antagonists, no significant change in the EPSP was observed. (Fig. 6A: 5-HT + 5-HT_1A/2/A/7_ antagonists, 10 cells, 95.13 ± 38, p = 0.930 based on a Wilcoxon signed rank test). As shown in Fig. 6B, the long-term effect of TBS was not significantly different when compared with control slices (Fig. 6B: p = 0.114 on a Mann–Whitney U Test test). We next studied the probability distribution of plasticity events induced by TBS in slices treated with 5-HT and 5-HT_1A/2A/7_ antagonists. We found that most cells achieved LTD with only a relative minority exhibiting LTP. This distribution of events is not significantly different when compared with the control situation (Fig. 6C: p = 0.543 on a Chi squared test). In both cases, a higher probability of LTD expression is present. However, this event distribution is not translated into significant depression when the mean synaptic change values are calculated.

### Modulation of synaptic plasticity in P14-16 animals by 5-HT_1A/2A/7_ agonists

**Figure 7:**
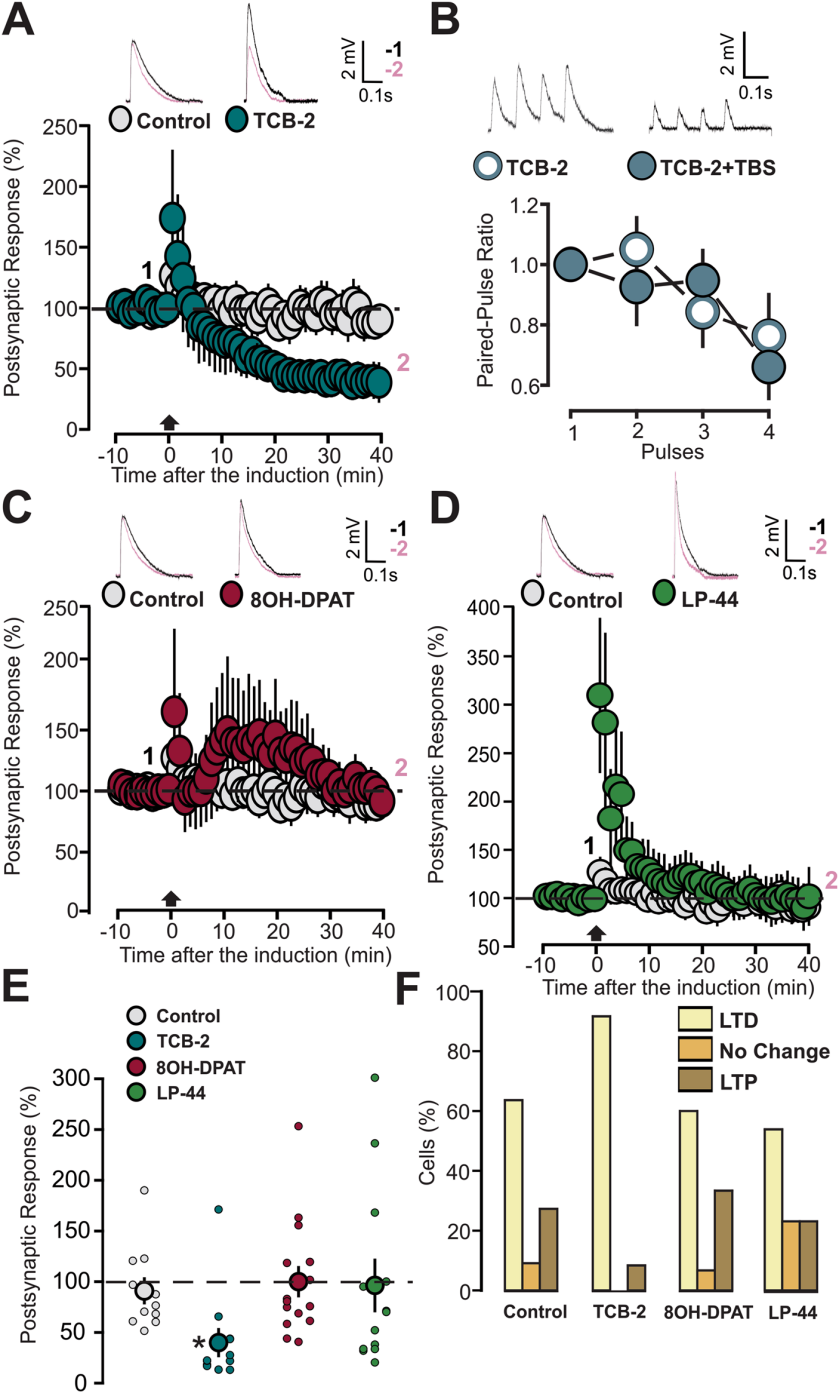
TBS coupled to activation of 5-HT_2A_ receptor induces LTD. (**A**) The graph shows the EPSPs recorded in P14-16 animals before and after TBS induction. Two different conditions are compared: control (light grey inserts) and application of 5-HT_2A_ receptor agonist (blue inserts, TCB-2, 10 µM). The EPSPs were normalized to the mean of responses recorded during the baseline. Traces show the results of a representative experiment with the average of postsynaptic responses recorded before (1: baseline, − 5 to 0 min, black line) and after the TBS induction (2: post induction, 35–40 min, black line). (**B**) Graph showing the paired pulse ratio of 4 pulses (10 Hz) EPSPs in slices treated with TCB-2. Two different conditions are compared: before (white inserts) and after TBS induction (blue inserts). Postsynaptic responses were normalized to the response caused by the first pulse. Traces show the results of a representative experiment with the average of postsynaptic responses recorded before the baseline and 40 min after the TBS induction. (**C**) The graph shows the EPSPs recorded in P14-16 animals before and after TBS induction. Two different conditions are compared: control (light grey inserts) and application of 5-HT_2A_ receptor agonist (red inserts, 8-OH-DPAT, 1 µM). The EPSPs were normalized to the mean of responses recorded during the baseline. Traces show the results of a representative experiment with the average of postsynaptic responses recorded before (1: baseline, − 5 to 0 min, black line) and after the TBS induction (2: post induction, 35–40 min, black line). (**D**) The graph shows the EPSPs recorded in P14-16 animals before and after TBS induction. Two different conditions are compared: control (light grey inserts) and application of 5-HT_2A_ receptor agonist (red inserts, LP-44, 2 µM). The EPSPs were normalized to the mean of responses recorded during the baseline. Traces show the results of a representative experiment with the average of postsynaptic responses recorded before the average of postsynaptic responses recorded before (1: baseline, − 5 to 0 min, black line) and after the TBS induction (2: post induction, 35–40 min, black line). (**E**) Scatter graph showing the averaged EPSPs recorded during the 35–40 min period after TBS induction, for individual experiments (small dots) and for the mean values of the experimental group (large dots, with bars representing the SEM). Five experimental groups are compared based on the condition of drug incubation (Control, TCB-2, 8-OH-DPAT and LP-44). EPSPs values were normalized to the mean of responses recorded during the baseline. (**F**) The graph shows the percentage of cells exhibiting LTP, LTD or no change in five experimental groups (Control, TCB-2, 8-OH-DPAT and LP-44).

We next studied the effects of 5-HT activity modulation on TBS-induced plasticity in P14-16 animals, using 5-HT_1A/2A/7_ receptor agonists. The induction applied to slices under the effect of the 5-HT_2A_ receptor agonist TCB-2 (10 µM) caused an LTD (Fig. 7A: 12 cells, 39.9 ± 12.7, p = 0.006 on a Wilcoxon signed rank test; Supplementary Figure C: Slope, Control: 10 cells, 112.15 ± 15.6, p = 0.364, on a paired t-testTCB-2: 12 cells, slope, 42.2 ± 13.2, p = 0.0098 on a Wilcoxon signed rank test). We performed an analysis of PPR to test the possibility of a presynaptic component, by applying a four-pulse train (10 Hz). By comparing the PPR in the two conditions (before and after TBS induction), no significant difference was found (Fig. 7B: 11 cells; baseline 0.76 ± 0.14; TCB-2 0.66 ± 0.10, p = 0.201 on a paired t-test). Investigation of the TBS-induced plasticity under the modulation of either the 5-HT_1A_ (8-OH-DPAT, 1 µM) or 5-HT_7_ receptor agonist (LP-44, 2 µM) revealed that the high-frequency trains did not cause a significant modification of the EPSPs in both situation (Fig. 7C: 8-OH-DPAT, 15 cells, 99.9 ± 14.5, p = 0.997 on a paired t-test; Fig. 7D: LP-44, 14 cells, 96.3 ± 25.1, p = 0.509 on a Wilcoxon signed rank test).

Figure 7E summarizes the effects of the 5-HT_1A/2A/7_ agonists. Selective 5-HT_2A_ stimulation paired with TBS allowed the occurrence of LTD, while either 5-HT_1A_ or 5-HT_7_ agonists failed to promote TBS-induced plasticity. (Fig. 7E: p = 0.002 on a Kruskal Wallis test; p = 0.001 for TCB-2 versus Control; p = 0.0004 for TCB-2 versus 8-OH-DPAT; p = 0.013 for TCB-2 versus LP-44. All comparisons were done on a Dunn post hoc test).

Figure 7F shows the effects of 5-HT_1A/2A/7_ agonists in changing probability distribution of the plasticity events. 5-HT_1A/2A/7_ agonists did not change the relative proportion of cells undergoing the three forms of plasticity (Fig. 7F: p = 0.313 on a Chi squared test). This result indicates that in control situations TBS-induced synaptic depression in most cells, but this effect was small and not statistically significant. On the other hand, selective stimulation of the 5-HT_2A_ receptor facilitated the TBS-induced LTD as the whole population outcome, by increasing the quantitative impact of synaptic depression in the proportion of cells showing LTD.

**Figure 8:**
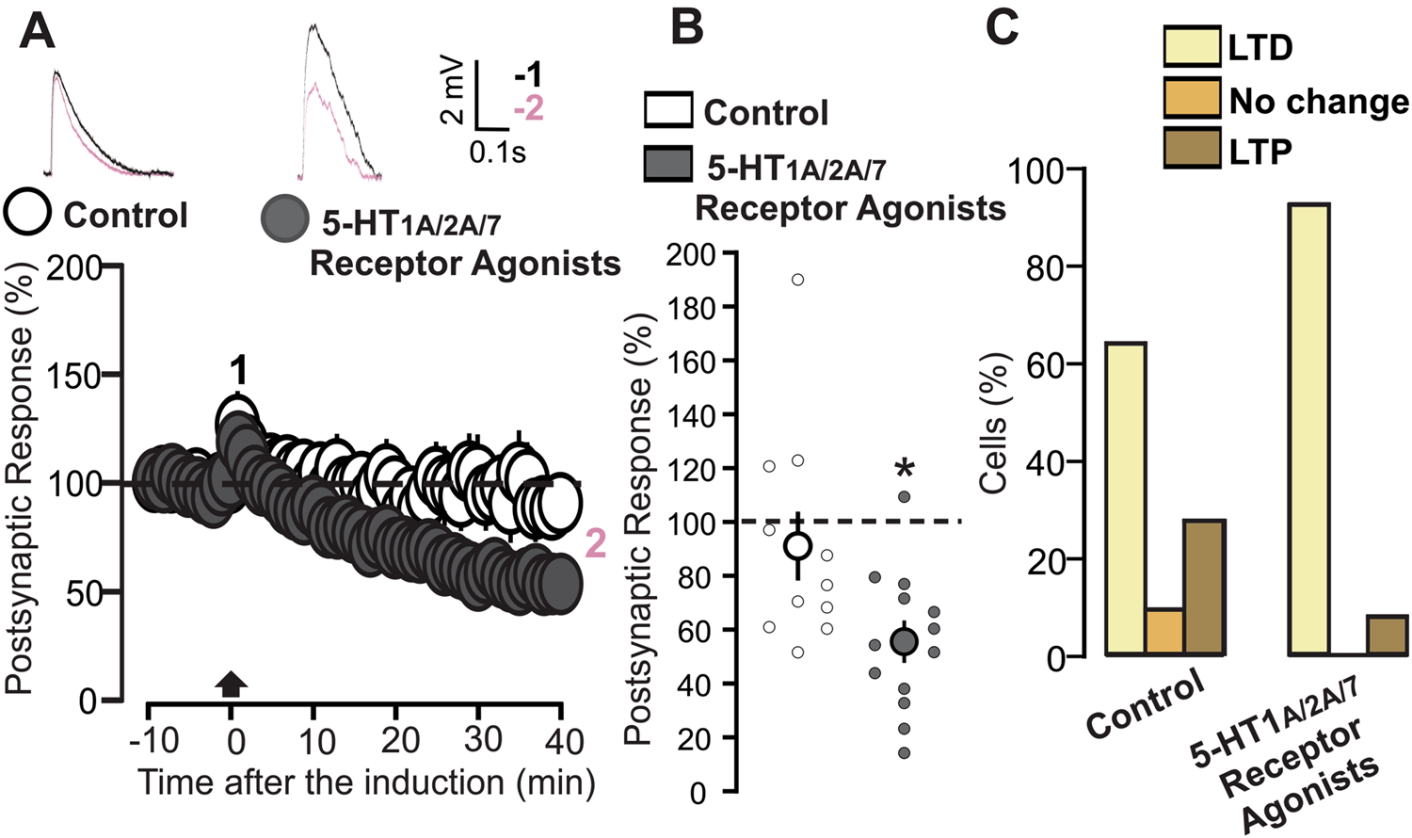
Application of three 5-HT_1A/2A/7_ receptors agonists induces LTD. (**A**) The graph shows the EPSPs recorded in P14-16 animals before and after TBS induction. The EPSPs were normalized to the mean of responses recorded during the baseline. Two different conditions are compared: control (white inserts) and 5-HT_1A/2A/7_ receptor agonists (dark grey inserts: TCB-2, 10 µM, 8-OH-DPAT, 1 µM LP-44, 2 µM). Traces show the results of a representative experiment with the average of postsynaptic responses recorded before (1: baseline, − 5 to 0 min, black line) and after the TBS induction (2: post induction, 35–40 min, black line). (**B**) Scatter graph showing the averaged EPSPs recorded during the 35–40 min period after TBS induction, for individual experiments (small dots) and for the mean values of the experimental group (large dots, with bars representing the SEM). Two experimental groups are compared based on the condition of drug incubation: control and 5-HT_1A/2A/7_ receptors agonists (TCB-2, 10 µM, 8-OH-DPAT, 1 µM LP-44, 2 µM). EPSPs values were normalized to the mean of responses recorded during the baseline. (**C**) The graph shows the percentage of cells exhibiting LTP, LTD or no change in two experimental groups: control and 5-HT_1A/2A/7_ receptors agonists (TCB-2, 10 µM, 8-OH-DPAT, 1 µM LP-44, 2 µM).

As a final step in our experiments with 5-HT_1A/2A/7_ agonists, we investigated whether the joint application of the three drugs mimics the effects of 5-HT. For this purpose, we designed a series of experiments in which slices were incubated along with the three 5-HT_1A/2A/7_ agonists together during the plasticity experiments. Under these conditions, the TBS produced an LTD which was similar to that observed previously under the influence of the 5-HT_2A_ agonist (Fig. 8A: 5-HT_1A/2/A/7_ agonists, 13 cells, 55.51 ± 7.19, p = 5.67 × 10^–5^ on a paired t-test). This synaptic change is significantly different when compared with the effect of TBS on control slices (Fig. 8B: p = 0.021 on a t-test). Finally, we studied the distribution of plasticity events for this set of experiments. As shown in the Fig. 8B, the vast majority of cells expressed LTD. This distribution is no different from the control situation (Fig. 8C: p = 0.205 on a Chi squared test). This suggests that agonists facilitate synaptic depression not by increasing probability of LTD occurrence but rather by increasing the amount of synaptic strength reduction in the LTD expression.

### 5-HT and 5-HT_1A/2A/7_ agonists do not change neuronal excitability in P14-16 animals

Changes in synaptic plasticity facilitated by serotonergic modulation may be indirectly caused by alterations in neuronal excitability induced by 5-HT. To investigate this possibility, we performed a series of experiments to study the effects of 5-HT and 5-HT_1A/2A/7_ receptors agonists on the membrane potential and the input resistance. 5-HT or one of the three 5-HT receptor agonists was applied after a 10 min baseline for a 30 min period. The mean of the baseline values (5–10 min) was compared with the mean of the values recorded under the effects of the drug (25–30 min).

**Figure 9:**
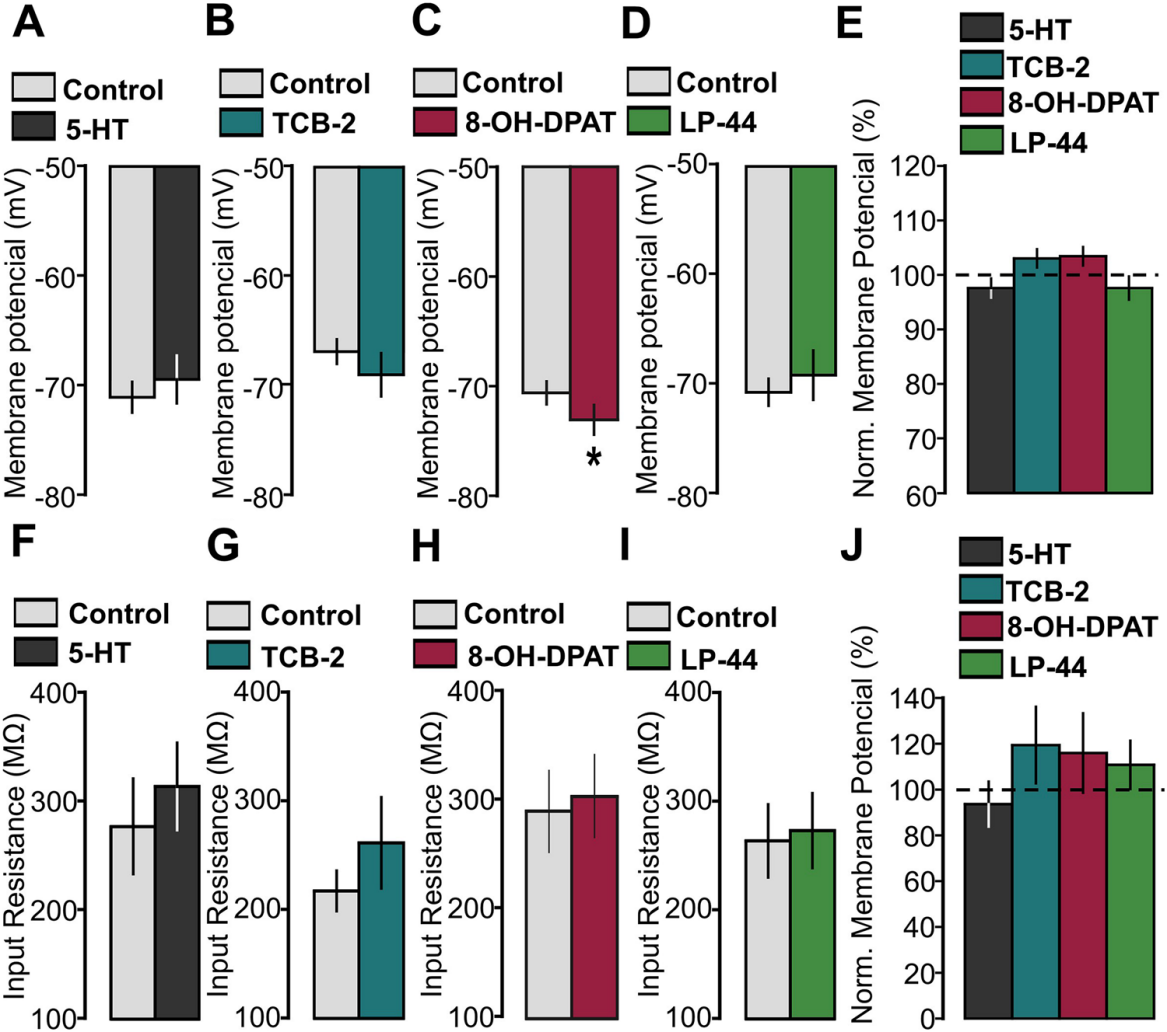
5-HT and the 5-HT_1A/2A/7_ receptors agonists have no effect on neuronal excitability. (**A**) Graph showing the averaged membrane resting potential recorded in the control condition (before the drug application, for 5 min) and under the effect of 5-HT (50 µM, from 25 to 30 min after the drug application). (**B**) Graph showing the averaged membrane resting potential recorded in the control condition (before the drug application, for 5 min) and under the effect of TCB-2 (10 µM, from 25 to 30 min after the drug application). (**C**) Graph showing the averaged membrane resting potential recorded in the control condition (before the drug application, for 5 min) and under the effect of 8-OH-DPAT (1 µM, from 25 to 30 min after the drug application). (**D**) Graph showing the averaged membrane resting potential recorded in the control condition (before the drug application) and under the effect of LP-44 (2 µM, from 25 to 30 min after the drug application). (**E**) Graph showing the averaged membrane resting potential recorded under the effect of 5-HT and 5-HT_1A/2A/7_ agonists (from 25 to 30 min after the drug application). Four experimental groups are compared: 5-HT (50 µM), TCB-2 (10 µM), 8-OH-DPAT (1 µM) and LP-44 (2 µM). EPSPs values were normalized to the mean of membrane resting potential values recorded before the drug application, for 5 min. (**F**) Graph showing the averaged input resistance recorded in the control condition (before the drug application, for 5 min) and under the effect of 5-HT (50 µM, from 25 to 30 min after the drug application). (**G**) Graph showing the averaged input resistance recorded in the control condition (before the drug application, for 5 min) and under the effect of TCB-2 (10 µM, from 25 to 30 min after the drug application). (**H**) Graph showing the averaged input resistance recorded in the control condition (before the drug application, for 5 min) and under the effect of 8-OH-DPAT (1 µM, from 25 to 30 min after the drug application). (**I**) Graph showing the averaged input resistance in the control condition (before the drug application, for 5 min) and under the effect of LP-44 (2 µM, from 25 to 30 min after the drug application). (**J**) Graph showing the averaged input resistance recorded under the effect of 5-HT and 5-HT_1A/2A/7_ agonists (from 25 to 30 min after the drug application). Four experimental groups are compared: 5-HT (50 µM), TCB-2 (10 µM), 8-OH-DPAT (1 µM) and LP-44 (2 µM). EPSPs values were normalized to the mean of input resistance values recorded before the drug application, for 5 min.

5-HT did not cause significant alterations in the resting membrane potential (Fig. 9A: n = 11, Control: − 71.04 ± 1.4, 5-HT: − 69.41 ± 2.18, p = 0.255 on a t-test). No changes of membrane potential value were observed in cells stimulated with the 5-HT_2A_ (TCB-2, 10 µM) or 5-HT_7_ (LP-44, 2 µM) receptor agonists (Fig. 9B: n = 12, Control: − 67.07 ± 1.13, TCB-2: − 69.22 ± 2.0, p = 0.303 on a t-test; Fig. 9D: n = 12, Control: − 70.87 ± 1.24, LP-44: − 69.29 ± 2.28, p = 0.305 on a t-test). On the other hand, the 5-HT_1A_ receptor agonist (8-OH-DPAT, 1 µM) produced a slight but significant hyperpolarization (Fig. 9C: n = 14, Control: -69.68 ± 0.99, 8-OH-DPAT: − 72.03 ± 1.29, p = 0.035 on a t-test). Figure 9E summarizes the effects of 5-HT and 5-HT_1A/2A/7_ receptors agonists on membrane potential. For each case, the membrane potential values were normalized with respect to the baseline values. Statistical analysis did not result in any significant differences (p = 0.087 on a Kruskal–Wallis test).

Bath administration of 5-HT had no significant effect on the input resistance (Fig. 9F: n = 11, Control: 276.34 ± 43.92, 5-HT: 313.27 ± 93.63, p = 0.319 on a t-test). Application of the 5-HT_2A_, the 5-HT_1A_ or the 5-HT_7_ receptor agonist also showed no significant change of input resistance (Fig. 9G: n = 12, Control: 214.38 ± 17.85, TCB-2: 257.5 ± 40.59, p = 0.529 on a Wilcoxon signed-rank test; Fig. 9H: n = 14, Control: 284.19 ± 35.28, 8-OH-DPAT: 297.23 ± 36.47, p = 0.341 on a t-test; Fig. 9I: n = 12, Control: 258.34 ± 32.22, LP-44: 267.5 ± 33.0, p = 0.329 on a t-test). Figure 9J shows the input resistance values for the four groups (5-HT, 5-HT_2A_ receptor agonist, 5-HT_1A_ receptor agonist and 5-HT_7_ receptor agonist), being normalized with respect to the respective baseline values. We did not find significant differences in the statistical analysis (p = 0.551 on a Kruskal–Wallis test).

### 5-HT activity modulation fails to facilitate synaptic plasticity in P24-26 animals

To test the hypothesis of the existence of an early age temporal window for 5-HT-dependent plasticity, we investigated the 5-HT modulatory effects on PFC plasticity on older animals (P24-26). We first examined the direct action of 5-HT on synaptic transmission.

**Figure 10:**
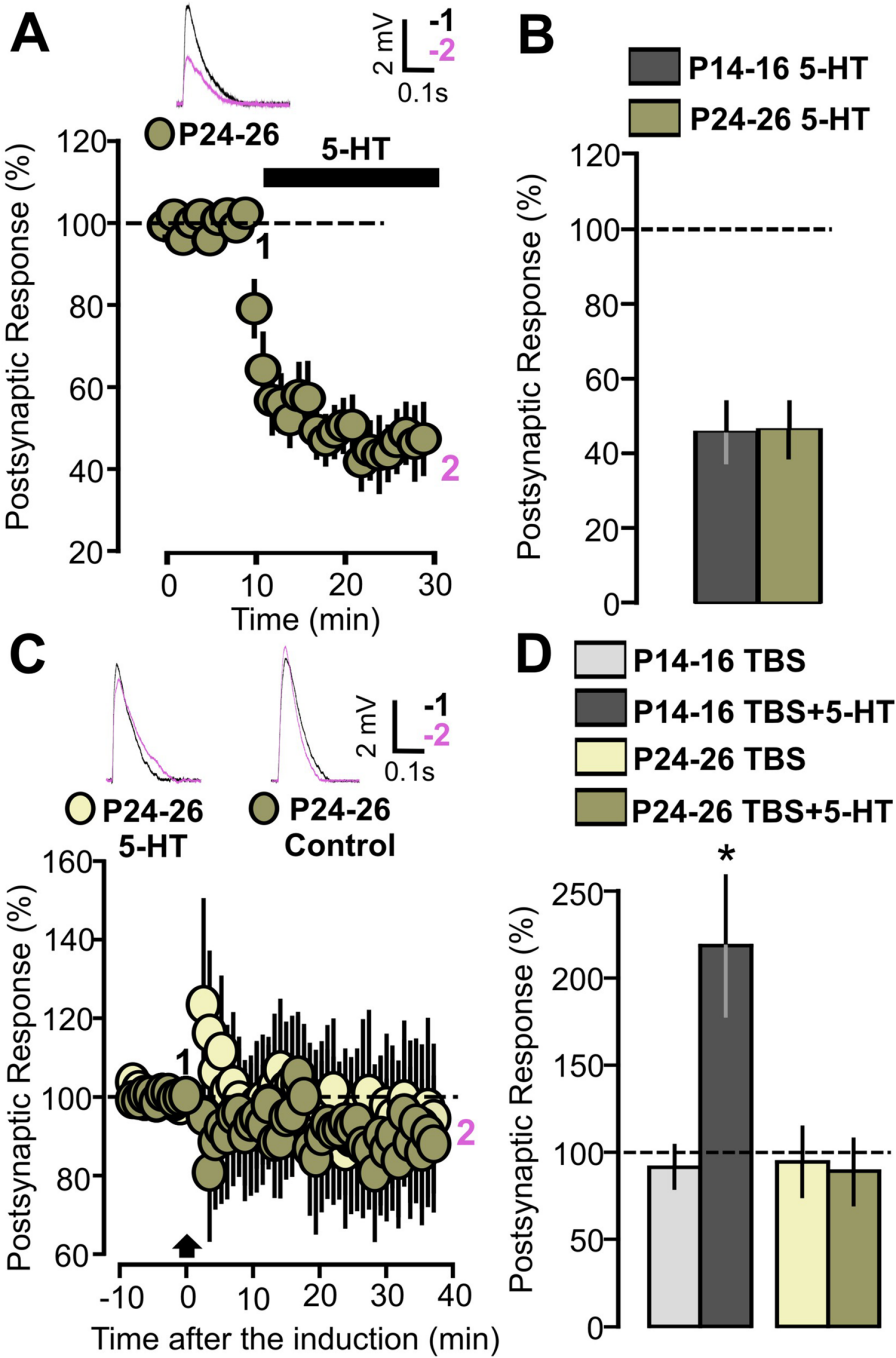
TBS induction fails to induce synaptic plasticity in slices from P24-26 animals. (**A**) The graph shows the EPSPs recorded in P24-46 animals before and during 5-HT bath application. The EPSPs were normalized to the mean of responses recorded during the baseline. The black bar represents the period of application of 5-HT (50 µM) in the bath. Traces show the results of a representative experiment with the average of postsynaptic responses recorded before (1: 5 to 10 min, black line) and after (2: 25 to 30 min, magenta line) application of 5-HT. (**B**) Graph showing the effects of 5-HT on the averaged post synaptic responses for the 25–30 min period in P14-16 and P24-26 animals. EPSPs were normalized to the mean of responses recorded during the baseline. (**C**) The graph shows the EPSPs recorded in P24-26 animals before and after TBS induction. The EPSPs values were normalized to the mean of responses recorded during the baseline, with two different conditions being compared: control (yellow inserts) and 5-HT (brown inserts, 50 µM). Traces show the results of a representative experiment with the average of postsynaptic responses recorded before (1: baseline, − 5 to 0 min, black line) and after the TBS induction (2: post induction, 35–40 min, black line). (**D**) Graph showing the averaged EPSPs recorded during the 35–40 min period after TBS induction. Four experimental groups are compared (P14-16 TBS, P14-16 TBS + 5-HT, P24-26 TBS, P24-16 TBS + 5-HT).

Bath application of 5-HT in P24-26 animals slices produced a 60% reduction of the EPSPs (Fig. 10A: 11 cells, 46.5 ± 7.5, p = 0.034, on a Wilcoxon signed rank test), thus indicating that the inhibitory effect of 5-HT on EPSPs is not age-dependent. This effect is not statistically different when compared with the 5-HT induced depression previously observed in younger animals (Fig. 10B: p = 0.342, on a Mann–Whitney U test). We next studied 5-HT modulation on TBS-induced synaptic changes. When the induction protocol was applied to control untreated slices, no significant difference in the EPSPs was found (Figurer 10C; 9 cells, 94.4 ± 20.0, p = 0.799, based on a paired t-test). When TBS had been delivered with the presence of 5-HT in the bath, no changes in postsynaptic responses were observed either (Figurer 10C; 10 cells, 89.2 ± 18.0, p = 0. 546, on a paired t-test).

Figure 10D summarizes the results of the 5-HT modulation of TBS-induced plasticity obtained from P14-16 and P24-26 animals. Younger animals exhibited a higher 5-HT dependent potentiation when compared to older animals (Fig. 10D: p = 0.015 on a two-way ANOVA).

### 5-HT fails to facilitate synaptic plasticity in MS animals

The development of PFC is highly sensitive to chronic stress in the postnatal period^37–41^. Maternal separation (MS) impairs 5-HT modulation of PFC activity during the early life and these effects cause behavioral modifications that persist until adult life^35^. MS-related changes of 5-HT signaling are mainly associated with reduction of 5-HT_2A_ receptor expression and with reduced activation of 5-HT_2A_ receptor-regulated intracellular pathways^42^. Based on these observations, we reasoned that, if 5-HT-dependent plasticity is important for PFC development, exposure to chronic stress in the early postnatal period should affect synaptic plasticity.

**Figure 11:**
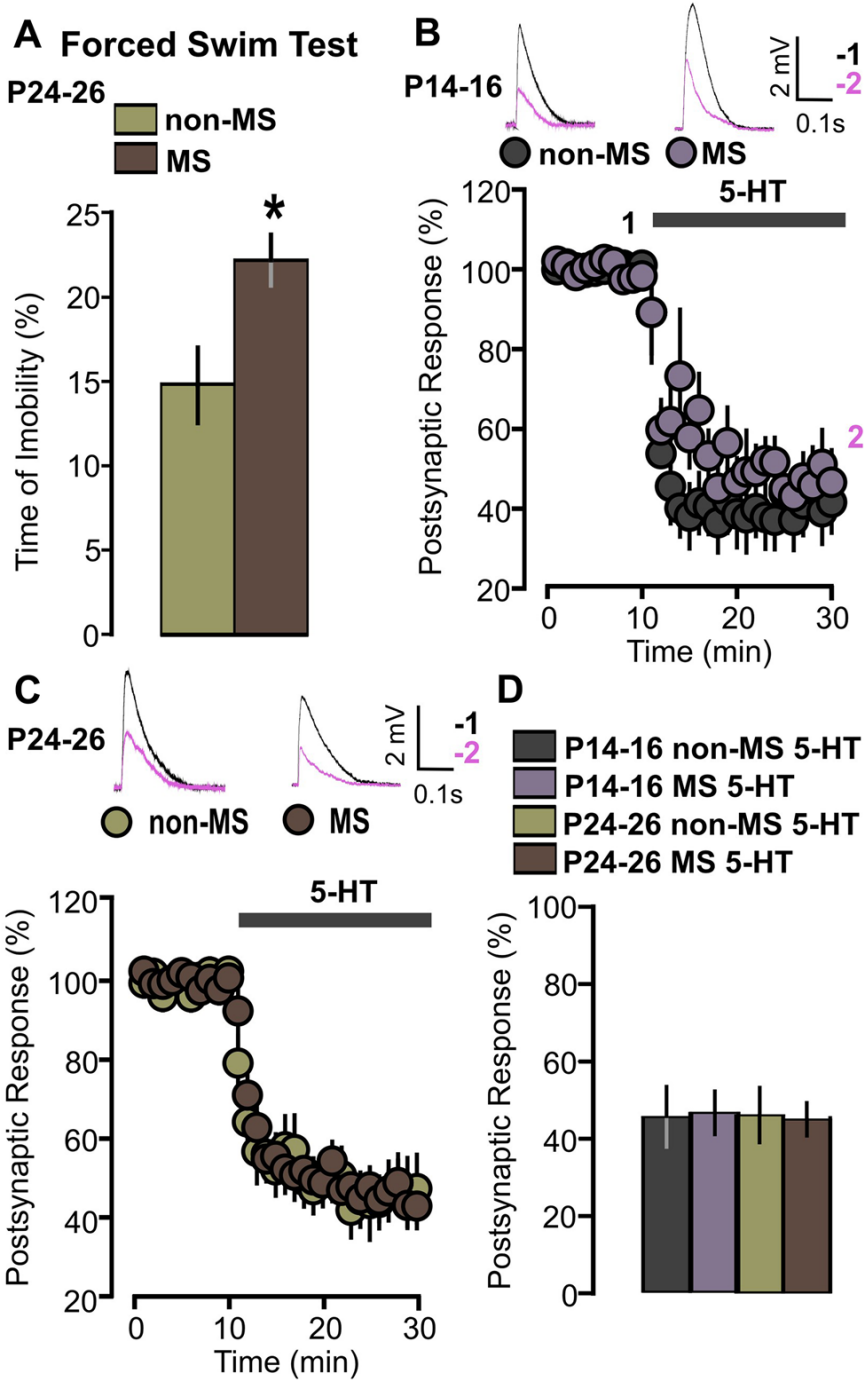
5-HT reduces the amplitude of postsynaptic responses in MS animals. (**A**) The graph shows the time of immobility measured in the Forced Swim Test performed on P24-26 animals. Two groups of animals are compared: MS and non-MS. Bars represent the average of normalized (%) time of immobility with SEM. (**B**) The graph shows the EPSPs recorded in P14-16 animals before and during 5-HT bath application. The EPSPs were normalized to the mean of responses recorded during the baseline. Two different conditions are compared: non-MS (dark grey inserts) and MS (light violet) animals. The black bar represents the period of application of 5-HT (50 µM) in the bath. Traces show the results of a representative experiment with the average of postsynaptic responses recorded before (1: 5 to 10 min, black line) and after (2: 25 to 30 min, magenta line) application of 5-HT. (**C**) Graph showing the effects of 5-HT on the averaged post synaptic responses recorded during the 25–30 min period in P14-16 MS animals. EPSPs were normalized to the mean of responses recorded during the baseline. (**D**) The graph shows the EPSPs recorded in P24-26 animals before and during 5-HT bath application. The EPSPs were normalized to the mean of responses recorded during the baseline. Two different conditions are compared: non-MS (brown inserts) and MS (dark brown inserts) animals. The black bar represents the period of application of 5-HT (50 µM) in the bath. Traces show the results of a representative experiment with the average of postsynaptic responses recorded before (1: 5 to 10 min, black line) and after (2: 25 to 30 min, magenta line) application of 5-HT. (**E**) Graph showing the effects of 5-HT on the averaged post synaptic responses for the 25–30 min period in P24-26 MS animals. EPSPs were normalized to the mean of responses recorded during the baseline.

Based on this hypothesis, we subjected new sets of animals to MS between 2 and 14 days. To ascertain whether the separation protocol effectively changed the behavioral profile of MS animals, two groups of P24-26 animals (MS and non-MS) were analyzed in the FST. We found that MS rats exhibit longer periods of immobility compared to non-separated (Fig. 11A: non-MS, 14.84 ± 2.17%, 12 animals; MS, 22.19 ± 1.40%, 13 animals, mean ± SEM, p = 0.007, on a t-test).

We next investigated the effects of 5-HT on the EPSPs of slices obtained from MS rats. In P14-16 MS animals, administration of 5-HT strongly reduced postsynaptic responses (Fig. 11B: non-MS, 10 cells, 45.8 ± 8.2, p = 5 × 10^–5^, on a paired t-test; MS P, 10 cells, 46.9 ± 5.9, p = 10^–6^, on a paired t-test), suggesting that 5-HT inhibits synaptic transmission in layer 5 of PFC regardless of the stress condition. Similar effects were found in P24-26 MS, where 5-HT strongly reduced the EPSP values (Fig. 11C: non-MS, 11 cells, 46.5 ± 7.5, p = 0.034, on a Wilcoxon signed rank test; MS, 15 cells, 47.6 ± 8.5, p = 6 × 10^–7^, based on a paired t-test). Figure 11D summarizes the results of the 5-HT modulation of EPSPs in non-MS and MS animals in the two age periods (P14-16 and P24-26). No significant difference was found in terms of age and separation condition (Fig. 11D: p = 0.746 and p = 0.709, respectively, on a two-way ANOVA).

**Figure 12:**
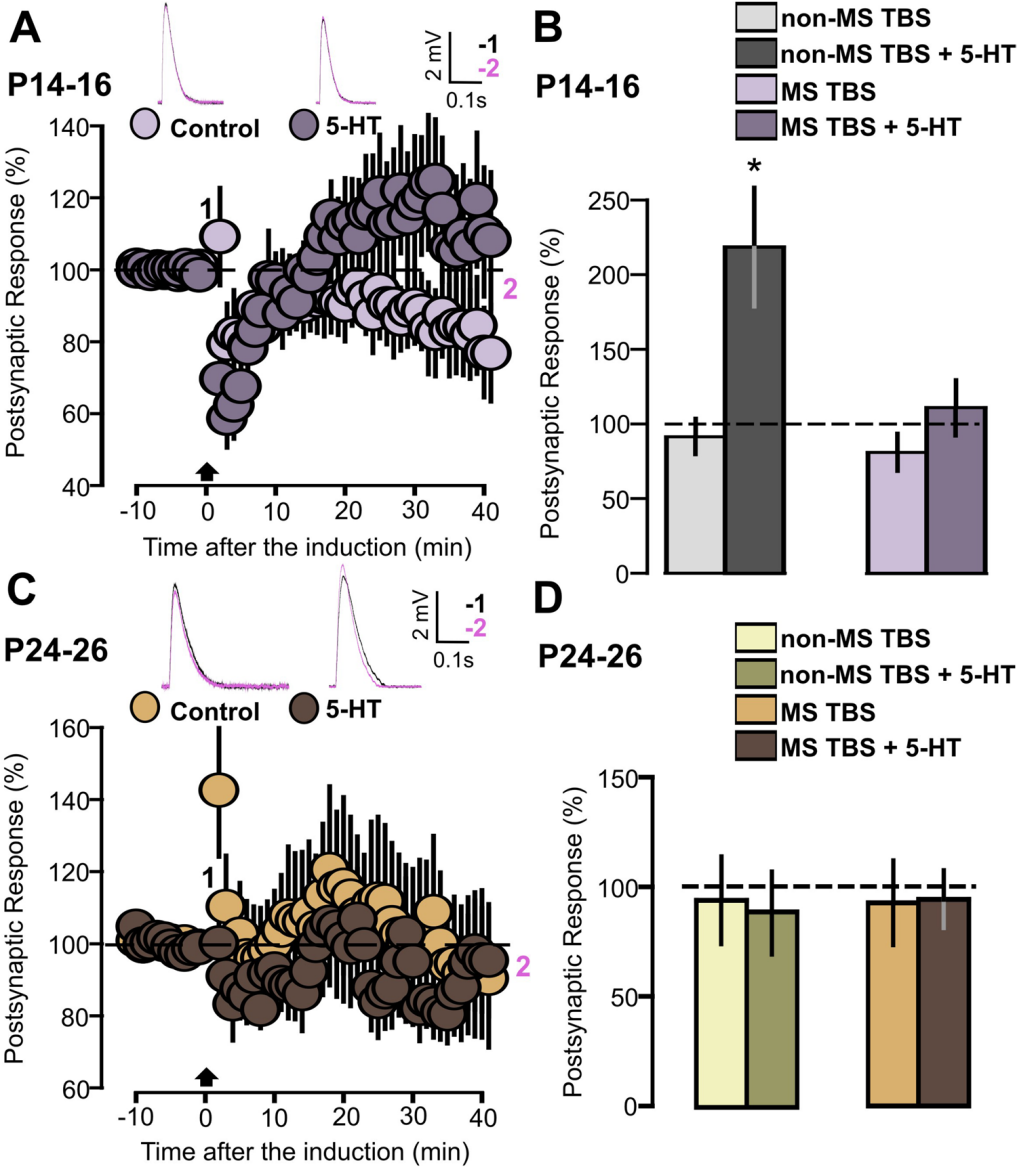
TBS induction fails to induce synaptic plasticity in slices from MS animals. (**A**) The graph shows the EPSPs recorded in MS P14-16 animals before and after TBS induction. The EPSPs were normalized to the mean of responses recorded during the baseline, with two different conditions being compared: control (light violet) and 5-HT (dark violet, 50 µM). Traces show the results of a representative experiment with the average of postsynaptic responses recorded before (1: baseline, − 5 to 0 min, black line) and after the TBS induction (2: post induction, 35–40 min, magenta line). (**B**) Graph showing the averaged EPSPs recorded during the 35–40 min period after TBS induction in P14-16 animals. Four experimental groups are compared (non-MS TBS, non-MS TBS + 5-HT, MS TBS, MS TBS + 5-HT). (**C**) The graph shows the EPSPs recorded in MS P24-26 animals before and after TBS induction. The EPSPs were normalized to the mean of responses recorded during the baseline, with two different conditions being compared: control (beige inserts) and 5-HT (dark brown inserts, 50 µM). Traces show the results of a representative experiment with the average of postsynaptic responses recorded before (1: baseline, − 5 to 0 min, black line) and after the TBS induction (2: post induction, 35–40 min, magenta). (**D**) Graph showing the averaged EPSPs recorded during the 35–40 min period after TBS induction in P24-26 animals. Four experimental groups are compared (non-MS TBS, non-MS TBS + 5-HT, MS TBS, MS TBS + 5-HT).

Finally, we tested the hypothesis that 5-HT-dependent synaptic plasticity could be reduced or absent in MS animals. The application of TBS on slices from P14-16 MS animals, without the presence of 5-HT, did not cause changes in EPSPs (Fig. 12A: Control, 11 cells, 80.9 ± 12.9, p = 0.174, based on a paired t-test). In slices treated with 5-HT and stimulated with TBS no changes in postsynaptic responses were found (Fig. 12A: 5-HT, 10 cells, 110.9 ± 18.5, p = 0.857, based on a Wilcoxon signed rank test), thus indicating that 5-HT fails to facilitate synaptic plasticity in MS animals. In Fig. 12B, we summarize our results about the interaction between MS and 5-HT-dependent plasticity in P14-16 animals. Synaptic potentiation caused by TBS and 5-HT is enhanced in non-MS when compared with MS animals (Fig. 12B: p = 0.035 on a two-way ANOVA).

These results open the possibility of a shift of the temporal window for synaptic changes, suggesting that, in MS animals, 5-HT-dependent plasticity could occur at older ages. To test this hypothesis, we studied the effects of 5-HT modulation on TBS-induced plasticity in P24-26 MS animals. However, also at this age, TBS was not effective in inducing synaptic changes and treatment with 5-HT did not significantly change this profile (Fig. 12C: Control, 15 cells, 92.7 ± 19.2, p = 0.472, on a Wilcoxon signed rank test; 5-HT, 14 cells, 94.3 ± 13.1, p = 0.332, on a Wilcoxon signed rank test). Figure 12D exhibits the effects of 5-HT-dependent plasticity in MS and non-MS P24-26 animals and shows that synaptic changes are not significantly affected by MS (Fig. 12D: p = 0.560 on a two-way ANOVA).

## Discussion

This study aimed to investigate the effects of 5-HT-evoked modulation on synaptic plasticity in layer 5 pyramidal neurons of the PFC during the early postnatal period. While 5-HT application depressed the efficacy of synaptic transmission, TBS did not cause significant modification of EPSPs. However, when TBS was delivered under 5-HT modulation, synaptic changes occurred, with the expression of LTP or LTD being dependent on the 5-HT receptors that were activated. These effects were restricted to P14-16 animals and were absent in MS rats. These findings indicate that 5-HT mediated plasticity is limited to a temporal window and is impaired when PFC maturation is affected by chronic stress exposure.

We initially studied the direct effect of 5-HT bath application on excitatory synaptic transmission. Our data indicate that 5-HT strongly and rapidly suppresses the EPSPs amplitudes. 5-HT decreases glutamatergic synaptic strength by activating 5-HT_1A_, 5-HT_2A_, and 5-HT_7_ receptors in PFC slices from P14-16 animals. This is consistent with gene expression and electrophysiological studies, showing that these receptors are highly expressed in PFC pyramidal cells between the second and third week of the postnatal period^29,33,41–45^. This is also consistent with other studies reporting that 5-HT_1A_, 5-HT_2A_, and 5-HT_7_ receptors stimulation directly depress PFC synapses^32,33,45,46^. 5-HT_1A_ receptors reduce AMPA-mediated currents through activation of PP1, which relies on the inhibition of PKA and CaMKII^34^. On the other hand, 5-HT_2A_ receptor-mediated reduction of postsynaptic responses requires PKC, which promotes the internalization of AMPA glutamate receptors^32^.

We found that TBS applied at superficial layers failed to induce synaptic changes at layer 5 neurons without the presence of 5-HT. In cortical areas, TBS can induce plasticity by providing calcium influx through NMDA receptors or through metabotropic glutamate receptors activation^45–49^. However, cortical synapses respond to electrical induction with limited effects, being the availability of neuromodulators a critical factor that enhances the possibility of synaptic modification^50,51^. Our findings demonstrate that this property also applies to PFC synapses and agree with previous studies on neuromodulation of plasticity in this cortical area^30,32,52^. Nevertheless, our data revealed a 5-HT facilitation of TBS-induced plasticity that is restricted to the early postnatal period. This is a relevant feature compared with other developing cortical areas, where TBS typically produces synaptic changes even without a neuromodulator^53,54^. In this sense, synaptic plasticity of developing PFC in early life is particularly sensitive to 5-HT neuromodulation.

Our PFC slices obtained from P14-16 animals showed a strong postsynaptic LTP when TBS was delivered under 5-HT stimulating effects. Indirect evidence in the literature suggests some intracellular processes triggered by 5-HT_1A_ and 5-HT_7_ receptors that might contribute to this LTP. In the PFC, 5-HT_1A_ receptors stimulate the PI3K/Akt signaling pathway, whose main target is the rapamycin complex (mTORC), a protein kinase involved in the translational mechanisms of long-term plasticity and synaptogenesis^53–57^. The temporal window of LTP coincides indeed with the time course of AKT phosphorylation^58,59^. Furthermore, in several brain structures, including the PFC, the activation of PI3K cascade is required for the expression and maintenance of synaptic strengthening^58–63^. On the other hand, 5-HT_7_ receptor activation promotes an increase in the inward current in layer 5 pyramidal neurons of the PFC during development and this increase in excitability could facilitate the efficacy of electrical induction of plasticity^29,45^. 5-HT_7_ receptors activate two different intracellular pathways, involving G_s_ and G_12_ proteins respectively^64^. The G_s_ cascade increases intracellular cAMP concentrations, leading to PKA activation, which is required for LTP in the PFC^65,66^. G_12_ protein signaling pathway activated by 5-HT_7_ receptors stimulates synaptogenesis in the hippocampus, being its involvement restricted to early development^64,67^. Layer 5 pyramidal neurons of PFC undergo intense formation of new synapses in the early postnatal period and this process could involve LTP mechanisms that require 5-HT_7_ receptors and G_12_ protein signaling^68^.

Interestingly, 5-HT exposure together with either 5-HT_2A_, 5-HT_1A_, or 5-HT_7_ receptor antagonists caused the TBS protocol to induce a postsynaptic LTD. Similar results were observed when electrical induction was coupled with 5-HT_2A_ receptor agonist. Thus, TBS associated with activation of 5-HT_2A_ receptor or with activation of both 5-HT_1A_ and 5-HT_7_ receptors induces LTD at layer 5 neurons. The involvement of 5-HT_2A_ receptor in promoting electrically induced LTD in PFC circuitry has been observed in other studies. 5-HT_2A_ receptors facilitate high-frequency induced LTD by cooperating with group 1 metabotropic glutamate receptors^32^. The intracellular response of 5-HT_2A_ receptors stimulation and LTD induced by high frequency stimulation converge to the same IP3/DAG/PKC pathway and to the phosphorylation of AMPA receptor at the same subunits^33,69,70^. Other studies suggest that the 5-HT_1A_ receptor might also contribute to LTD by inhibiting the cAMP-PKA signaling^30,71^. 5-HT_1A_ receptor activation causes downregulation of PKA, CaMKII and ERK, resulting in NMDA receptors calcium currents reduction and thus shifting synaptic plasticity orientation towards the expression of LTD.

In the experiments where we applied the 5-HT_1A/2A/7_ agonists together, we could not reproduce the 5-HT-induced effects on plasticity. This result indicates that the three agonists are unable to fully mimic the same serotonergic modulatory response. Although the 5-HT_1A/2A/7_ types are the serotonergic receptors most involved in the modulation of PFC neural activity during postnatal development, we cannot exclude the participation of other receptor types^29^. In particular, 5-HT_3_ receptors have aroused increasing interest for their role in the modulation of cortical activity^72^. Being expressed in mature inhibitory GABAergic interneurons, 5-HT_3_ receptors fine-tune neuronal excitability and may possibly affect excitatory synaptic plasticity indirectly^72^. However, at P14-16, when we observed significant 5-HT-dependent synaptic changes, GABAergic interneurons are still relatively immature^73^. Furthermore, our results did not indicate any significant alterations in neuronal excitability caused by 5-HT at this age.

Another possible explanation lies in the limited pharmacological efficacy and selectivity of 5-HT_1A/2A/7_ receptors agonists and antagonists to their receptor subtype target. They primarily bind 5-HT receptors, but they can partially interact with other receptor types, especially if used at high concentrations^74^. TCB-2, LP-44 and SB-269970 have been reported to bind with high-affinities to their receptor targets^73–78^. On the other hand, 8-OH-DPAT, Ketanserin and WAY-100635 were found to also interact with other receptor types. WAY-100635 was originally thought as a selective 5-HT_1A_ receptor antagonist, but more recent studies have shown it also acts as a D4 receptor agonist. Nevertheless, expression of D4 receptors is low in the PFC, and their presence is even lower during the early postnatal period^79,80^. 8-OH-DPAT was found to be a partial agonist of 5-HT_7_ receptors, while ketanserin can partially function as 5-HT_2C_ antagonist^81,82^. Taken together, these considerations acknowledge that the efficacy of 5-HT_1A/2A/7_ receptors agonists and antagonists may be partial in some experimental situations. This may explain the relative difficulty that 5-HT_1A/2A/7_ agonists and antagonists might have had in respectively mimicking blocking and the 5-HT response completely.

**Figure 13:**
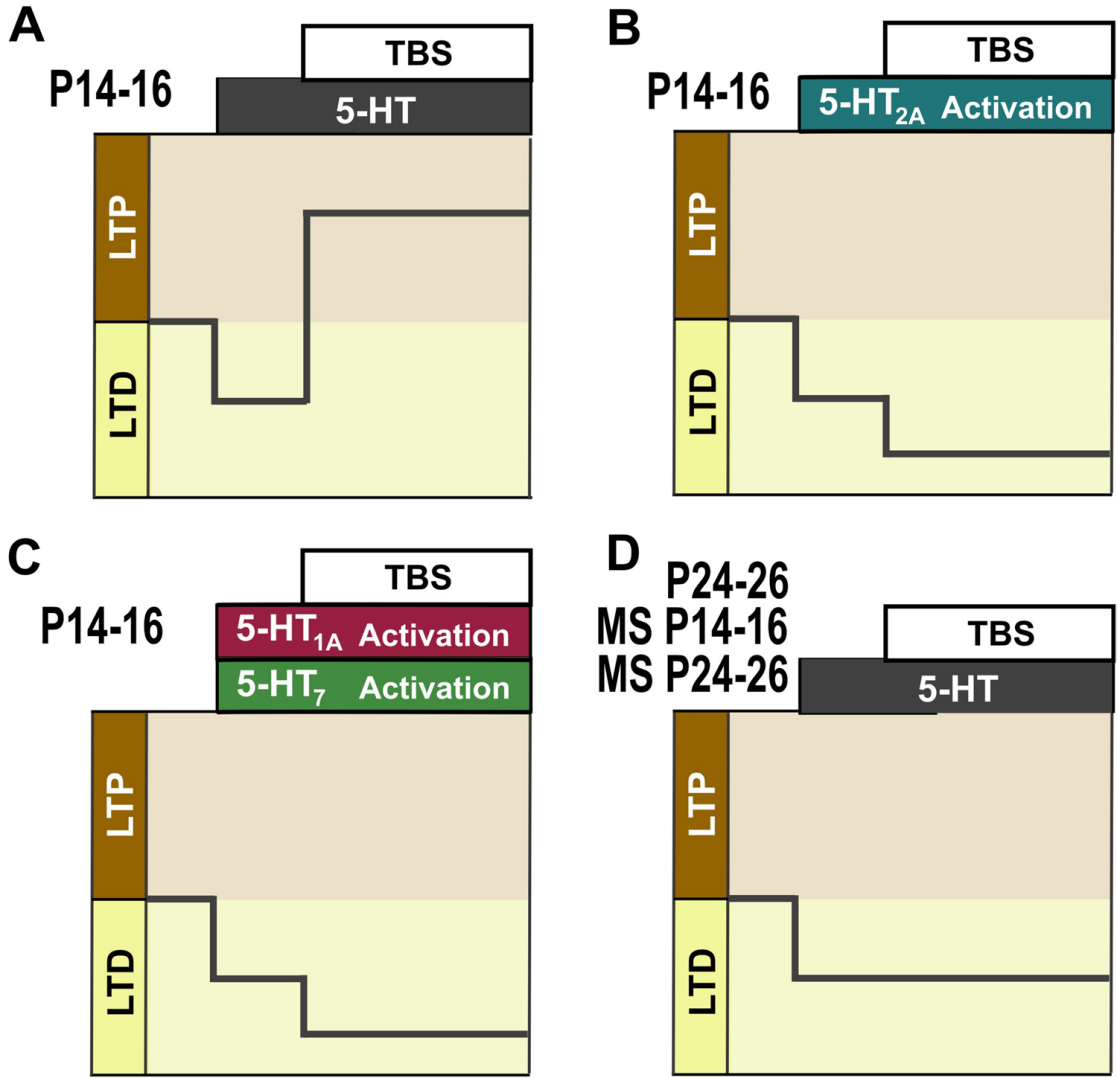
5-HT Modulation of Synaptic Plasticity. The figure summarizes the model proposed for the 5-HT modulation of synaptic plasticity induced by TBS. Limited 5-HT receptor types stimulation result in LTD expression, while the synergistic recruitment of multiple 5-HT receptor types activation allows the occurrence of LTP. The metaplastic interaction between TBS and 5-HT is age dependent and absent in the P24-26 period. (**A**) The expression of LTP requires the interaction between TBS and 5-HT. (**B**) TBS induction coupled with activation of 5-HT_2A_ receptor decreases the synaptic strength. (**C**) TBS induction coupled with activation of the 5-HT_1A_ and 5-HT_7_ receptors decreases the synaptic strength. (**D**) TBS induction coupled with 5-HT do not cause changes of synaptic efficacy in P24-26 animals.

Our results did not show significant effect of 5-HT or 5-HT_1A/2A/7_ on neuronal excitability, thus suggesting that TBS interacts with the 5-HT-induced depression to produce a direct metaplastic effect. The new direction of synaptic plasticity depends on the level of synergic cooperation of multiple 5-HT receptors. Figure 13 illustrates our model. If multiple 5-HT receptors are stimulated synergistically, synaptic change is directed towards the LTP (Fig. 13A). When 5-HT action is restricted to limited 5-HT receptor type activation (Fig. 13B, C), plasticity is rather directed towards LTD. In particular, stimulation of 5-HT_2A_ receptors paired with TBS is sufficient to induce synaptic depression (Fig. 13B). These metaplastic effects are age dependent and restricted to a temporal window of postnatal development, given that TBS combined with 5-HT fails to cause changes in synaptic strength at P24-26 (Fig. 13D).

5-HT_1A/2A/7_ receptors activate a complex intracellular response of multiple signaling pathways, involving the PKA, the DAG/IP3 and the PI3K/Akt cascade. It is possible to speculate that LTP expression would require the calcium influx provided by TBS to be accompanied by the synergic stimulation of the three intracellular pathways, being these activated by the neuromodulation provided by multiple 5-HT receptors. This view is also supported by previous studies showing that 5-HT_1A_ receptors may lead to opposite synaptic changes, depending on their interaction with intracellular cascades activated by other metabotropic receptors^31^. These conclusions suggest that changes in the relative expression of 5-HT receptors early in life can affect the gain of synaptic plasticity and, consequently, the reorganizing processes of PFC circuits during postnatal development. In accordance with this view, several studies associate the onset of neuropsychiatric disorders with impaired PFC plasticity and alterations in the expression of 5-HT receptors^83^.

Our data indicate the presence of a 5-HT-dependent plasticity in the PFC at P14-16 but not at P24-26. Although these results suggest a probable critical period for this form of plasticity restricted to early ages, they do not claim to define a critical window for plastic changes in general. Critical periods of modification of synaptic plasticity vary depending on the type of plasticity and on the different brain areas being considered. It is in fact known that the hippocampus and other cortical regions of the forebrain, as long as other forms of plasticity, also exhibit critical windows in later periods^82–87^. Being so, the relationship between age and synaptic plasticity needs to be integrated with other findings in order to obtain a more comprehensive picture.

Finally, we adopted a MS model to evaluate in vivo implications of our findings. Stress exposure during early life is known to modify serotonergic regulation of PFC in postnatal development, by changing synaptic transmission in pyramidal neurons^32,83,86–90^. Maternal separation disturbs the serotonergic system during early brain development by reducing mRNA expression coding for 5-HT_1A_ and 5-HT_2A_ receptors^35^. Moreover, MS reduces the 5-HT_2A_ receptor intracellular response and modifies the pattern of 5-HT_2A_ receptor regulated immediate early genes. These changes are associated with altered 5-HT_2A_ receptor-evoked behavioral response and they persist until adulthood^42^.

Based on this premise, we reasoned that, if the 5-HT dependent plasticity we investigated here were an essential physiological mechanism involved in PFC functional maturation, MS should affect it negatively. Our results confirm this hypothesis and show that stress impairs 5-HT-facilitated long-term plasticity in PFC. Interestingly, MS impairs 5-HT-dependent plasticity in P14-16 animals and produces effects similar to those obtained from unseparated P24-26 animals. This observation suggests that exposure to chronic stress in early life might induce accelerated maturation of PFC circuitry. This process would close the temporal opportunity for plasticity in advance, thus preventing the PFC from using a wider time window of environmental stimuli to perform its maturation.

## Supplementary Information

Supplementary Information.

## Data Availability

The data that support the findings of this study are available from Guilherme Shigueto Vilar Higa, but restrictions apply to the availability of these data, which were used under license for the current study, and so are not publicly available. Data are however available from the authors upon reasonable request and with permission of Guilherme Shigueto Vilar Higa.
