# Supplementary material for: 5-HT-dependent synaptic plasticity of the prefrontal cortex in postnatal development
Source: Sci Rep. 2022 Dec 5;12:21015. doi: 10.1038/s41598-022-23767-9 (PMC9723183; doi:10.1038/s41598-022-23767-9)
Supplement: Supplementary file 1 — Supplementary Information. [file 41598_2022_23767_MOESM1_ESM.pdf]

Title:

5-HT-dependent synaptic plasticity of the prefrontal cortex in postnatal development.

Author names and affiliations: Guilherme Shigueto Vilar Higa[1,2,4], José Francis-Oliveira[1 †], Estevão Carlos Lima[1 †], Alicia Moraes Tamais[1], Fernando da Silva Borges[3], Alexandre Hiroaki Kihara[2], Ianê Carvalho Shieh[1], Henning Ulrich[4], Silvana Chiavegatto[5,6], Roberto De Pasquale[1].

†: Equal Contribution

1: Laboratório de Neurofisiologia, Departamento de Fisiologia e Biofísica, Universidade de São Paulo, Butantã, São Paulo, SP 05508-000, Brasil

2: Laboratório de Neurogenética, Universidade Federal do ABC, São Bernardo do Campo, SP 09210-580, Brasil

3: Department of Physiology & Pharmacology, SUNY Downstate Health Sciences Brooklyn, NY 11203, US

4: Departamento de Bioquímica, Instituto de Química (USP), Butantã, São Paulo, SP 05508-900, Brasil

5: Laboratório de Neurociência Comportamental e Molecular, Departamento de Farmacologia, Instituto de Ciências Biomédicas (ICB), Universidade de São Paulo (USP), Butantã, São Paulo, SP 05508-000, Brasil

6: Departamento de Psiquiatria, Instituto de Psiquiatria do Hospital das Clínicas da Faculdade de Medicina da Universidade de São Paulo (HCFMUSP), São Paulo, SP 05508-903, Brasil

Supplementary Figure 1

1A: The graph shows the slope of the EPSPs recorded in P14-16 animals before and during 5-HT (50 µM) bath application. The slope values are normalized to the mean of responses recorded during the baseline and the bars are respect to the SEM. The black bar represents the period of application of 5-HT in the bath. **1B:** The graph shows the slope of the EPSPs recorded in P14-16 animals before and after TBS induction in the presence or not of 5-HT. The slope values are normalized to the mean of responses recorded during the baseline. Two different conditions are compared: control (white insert) and 5-HT (dark gray inserts, 50 µM). **1C:** The graph shows the slope of the EPSPs recorded in P14-16 animals before and after TBS induction under the application of $5\text{-}HT_{2A}$ receptor agonist (TCB-2, 10 µM). Two different conditions are compared: control (white insert) and TCB-2 (blue inserts, 10 µM).The slope values are normalized to the mean of responses recorded during the baseline.

Figure 1

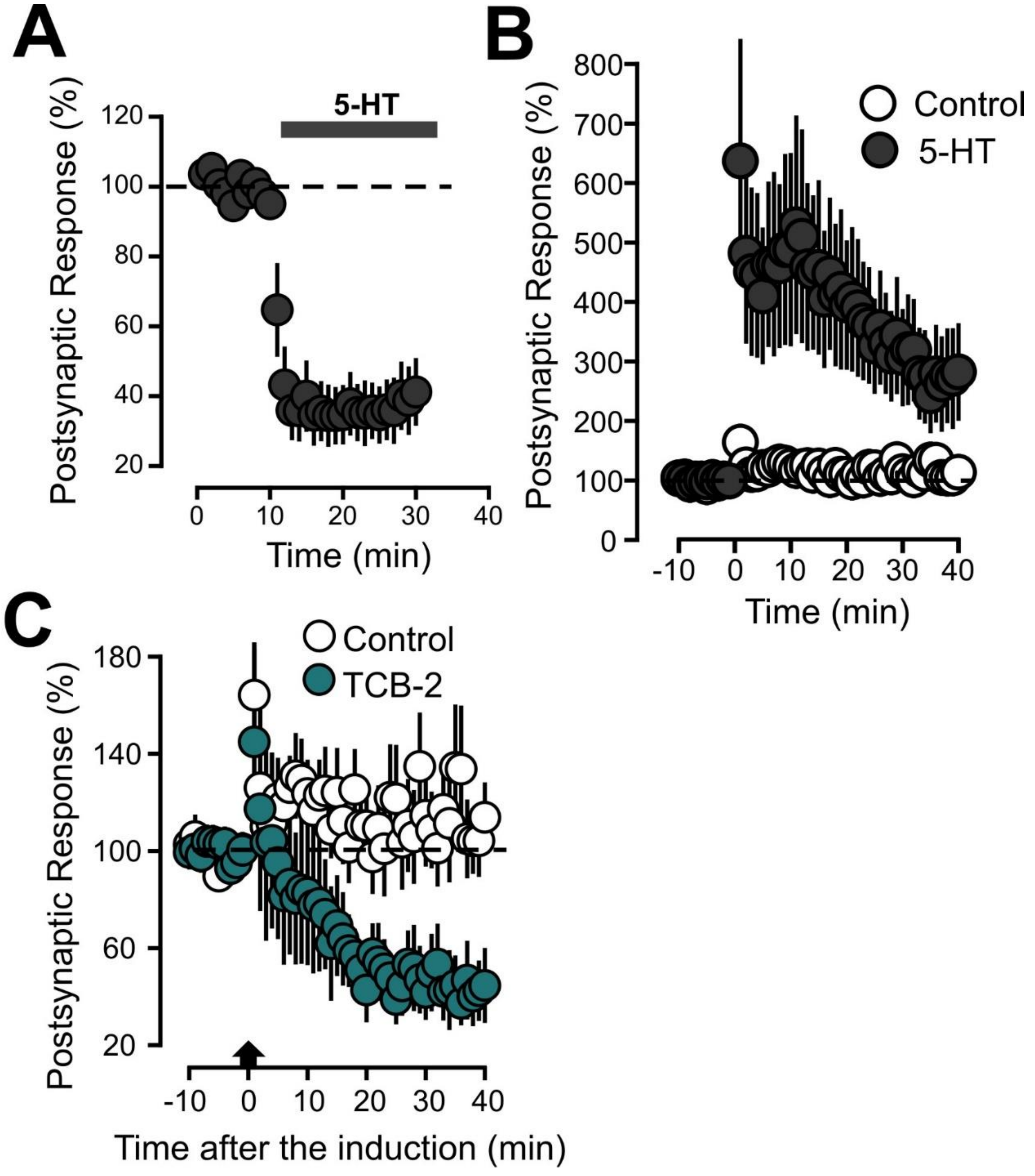
A
5-HT
Postsynaptic Response (%)
120
100
80
60
40
20
0
10
20
30
40
Time (min)
B
800
700
600
500
400
300
200
100
0
Control
5-HT
Postsynaptic Response (%)
-10
0
10
20
30
40
Time (min)
C
Control
TCB-2
180
140
100
60
20
Postsynaptic Response (%)
-10
0
10
20
30
40
Time after the induction (min)
